# The Effects of Social Media on Adolescent Mental Health: Findings From a Population‐Based Cohort Study in Australia

**DOI:** 10.5694/mja2.70220

**Published:** 2026-06-11

**Authors:** Nandita Vijayakumar, S. Ghazaleh Dashti, Louise Canterford, Susan Ellul, Anthony J. A. Parissi, Anne‐Lise Goddings, Russell M. Viner, Paul Moran, Rohan Borschmann, Lisa K. Mundy, Ellie M. Robson, Susan M. Sawyer

**Affiliations:** ^1^ Centre for Adolescent Health Royal Children's Hospital and Murdoch Children's Research Institute Melbourne Victoria Australia; ^2^ School of Psychology, Faculty of Health Deakin University Melbourne Victoria Australia; ^3^ Clinical Epidemiology and Biostatistics Unit Murdoch Children's Research Institute Melbourne Victoria Australia; ^4^ Department of Paediatrics The University of Melbourne Melbourne Victoria Australia; ^5^ Great Ormond Street Institute of Child Health University College London London England UK; ^6^ Department of Brain Sciences Imperial College London London England UK; ^7^ Centre for Academic Mental Health, Medical School University of Bristol Bristol England UK; ^8^ Department of Psychiatry, Medical Sciences Division University of Oxford Oxford England UK

**Keywords:** adolescence, longitudinal studies, social media

## Abstract

**Objectives:**

To examine the effects of social media on future mental health problems (depressive symptoms, anxiety symptoms, poor well‐being and self‐harm) in adolescents aged 12–18 years, overall and stratified by sex and age periods (early, middle and late adolescence).

**Study Type:**

Prospective longitudinal study.

**Setting:**

Participants were recruited in 2012 through schools in Melbourne, selected using stratified random sampling. In wave 1 (2012), 1239 Grade 3 students participated and have since completed annual surveys.

**Participants:**

The analysis used data up to wave 11 (2022). Participants with no data on mental health, social media and confounders were excluded, leaving a sample of 1195 (552 [46%] male participants).

**Main Outcome Measures:**

Exposure was self‐reported duration of daily social media use at each wave, from waves 4 to 10 (ages 12–18 years). Outcomes (self‐reported depressive symptoms, anxiety symptoms, well‐being, self‐harm) were assessed at the subsequent annual wave, from waves 5 to 11 (ages 13–19 years).

**Results:**

Across adolescence, > 2 h versus < 1 h of daily social media use was associated with a small increase in risk of high depressive symptoms (risk difference [RD] per 100, 6.3 [95% CI, 2.7–9.9]) and poor well‐being (RD, 4.9 [95% CI, 1.1–8.6]) at the subsequent annual wave. Estimated risks for all mental health problems were greatest in early adolescence (12–13 years), with the largest effects observed for high depressive symptoms in female participants (> 2 h vs. < 1 h: RD, 10.8 [95% CI, 2.7–18.9]).

**Conclusions:**

Higher levels of social media use were associated with small increases in future risk of high depressive symptoms and poor well‐being across adolescence. The largest risks for all mental health problems were observed during early adolescence for both male and female participants, supporting the need to consider policies that mitigate the adverse effects of social media on the mental health of younger adolescents.

## Plain Language Summary

**The Known:** Concerns about social media's impact on adolescent mental health have driven policy debate, yet evidence remains limited and largely cross‐sectional.

**The New:** Using annual data from a longitudinal cohort of Australian adolescents, we estimated future mental health risks from social media use between 12 and 18 years of age. More than 2 h of daily social media use was associated with an increased risk of high depressive symptoms and poor well‐being 1 year later. Estimated risks were greater in early adolescence (12–13 years) compared to risks in later ages.

**The Implications:** Early adolescence is a key vulnerability period for social media use, supporting targeted education and policies aimed at limiting use.

## 1 Introduction

Social media has transformed adolescents' lives by providing a platform for instantaneous and constant connectivity to peers. Many adolescents report positive experiences with social media [1], which may help foster key elements of psychosocial development, including social belonging, identity development and self‐expression [1]. However, high levels of adolescent mental health problems have sparked widespread concerns about the negative impacts of social media. Social media combines user‐generated content with engagement‐optimising algorithms, raising concerns about potential mental health impacts through mechanisms such as idealised social comparisons, cyberbullying and other negative peer interactions, emotionally salient content and displacement of sleep or offline activities. This has evoked calls for stricter regulations to mitigate potential harms [2], with many governments discussing or enacting policies to raise the minimum age to access social media [3, 4]. Notwithstanding public and political interest, there is little robust scientific evidence available to inform age‐based policy decisions.

Several reviews and meta‐analyses have reported weak or inconsistent associations between adolescents' social media use (SMU) and mental health, including for depression and poor well‐being [5, 6, 7, 8, 9]. These have noted poor methodological quality of studies, including an early focus on ‘screentime’ that did not distinguish different types of digital screen engagement, and a heavy reliance on cross‐sectional data that limits inferences regarding the temporality of associations. Although longitudinal studies have increasingly examined associations between SMU and future mental health [10], many have wide age bands at each wave, which can introduce cohort effects [11, 12, 13], or a limited number of waves that capture narrow developmental periods [14, 15]. Moreover, longitudinal studies have predominantly focussed on within‐subject effects of social media [16, 17, 18, 19, 20, 21, 22], the results of which are not easily translated into population‐level policy recommendations, or relied on structural equation models that cannot always be interpreted causally [23].

Studies have also primarily focussed on the effects of social media in adolescents as a homogenous group [5, 7], but age and sex differences may contribute to improved understanding of its impact on mental health [17, 24, 25]. One UK study found that SMU in early adolescence (11–13 years) was related to poorer life satisfaction in female adolescents, while similar associations were evident in middle adolescence (14–15 years) in male adolescents [21]. Further research, including consideration of a broader set of mental health outcomes, could inform potential social media regulations.

We sought to address these issues in a longitudinal population‐based cohort of Australians followed annually from childhood into early adulthood. We used data from nine annual waves to examine the effect of SMU at any given wave on multiple mental health outcomes (namely depressive symptoms, anxiety symptoms, poor well‐being and self‐harm) 1 year later. We considered effects across adolescence (12–18 years) as well as by sex (males, females) and age periods (early, middle and late adolescence).

## 2 Methods

### 2.1 Study Sample

Data for this study were drawn from the Child to Adult Transition Study (CATS), an ongoing longitudinal cohort study that was assembled to be representative of Grade 3 students in Melbourne, Australia in 2012 [26]. A stratified random sample of 43 schools was selected (government, Catholic and independent school strata). From these schools, all Grade 3 students (*N* = 2289) were invited to participate (there were no inclusion or exclusion criteria), of whom 54% (*n* = 1239) were recruited into the study. The main reason for non‐recruitment was failure to return a consent form (refer to Figure S1 for a flowchart of participants from recruitment onwards). Details on the representativeness of the cohort are presented in the Supporting Information.

Participants were followed annually from wave 1 (mean [standard deviation (SD)] age, 9.0 [0.4] years; 2012) to wave 11 (mean [SD] age, 18.9 [0.4] years; 2022). Data collection occurred through self‐reported questionnaires in primary schools (waves 1 to 4), with the additional option of survey completion online, by post or by computer‐assisted telephone interview (waves 5 and 6). From wave 7 (age 15 years) onwards, data collection was primarily completed online. At waves 1 and 2, a parent of each participant also completed a brief survey.

Participants were included in the current study if they had data on exposure (SMU), outcome (mental health) and/or confounders (peer victimisation, parent support, sleep duration and physical activity) at any wave from 3 to 11. This resulted in an analytic sample of 1195 participants. Similarities in demographic characteristics to the full cohort are presented in Table S1. Further details on missing data of analytic variables at each wave are presented in Table S2.

For the current analyses, SMU at each wave from 4 (mean [SD] age, 11.9 [0.4] years) to 10 (mean [SD] age, 17.8 [0.4] years) was examined as the exposure, with mental health outcomes assessed at the subsequent wave (i.e., exposure at wave t preceding outcome at wave *t* + 1). Confounders were measured at study baseline or at the prior wave (*t* − 1), so they temporally preceded the exposure.

### 2.2 Measures

#### 2.2.1 Sex

Sex (female, male) was collected at wave 1 by parent report.

#### 2.2.2 SMU

At each wave from 3 to 10, participants self‐reported their duration of SMU for a normal school day using a 6‐point scale from ‘none’ to ‘4 or more hours’. Based on distributions at early and late waves (see Figure S2), responses were categorised into three groups of daily duration of use (< 1, 1–2, > 2 h), to ensure sufficient numbers within exposure groups across adolescence. Details on social media sites endorsed at each wave are presented in Figure S3.

#### 2.2.3 Mental Health Problems

From waves 3 to 11, self‐reported depressive symptoms over the past 2 weeks were measured using the 13‐item Short Mood and Feelings Questionnaire [27]. From waves 3 to 10, self‐reported anxiety symptoms over the past 2 weeks were measured using a shortened 8‐item Spence Children's Anxiety Scale [28, 29], and at wave 11 were measured using the 7‐item Generalised Anxiety Disorder assessment [30]. From waves 3 to 10, self‐reported subjective well‐being was measured using six items from the Paediatric Quality of Life Inventory General Well‐Being Scale [31]. Data from these measures were dichotomised to define high levels of depressive symptoms, high levels of anxiety symptoms and poor well‐being at each wave (see Table S3 for further detail). From waves 4 to 11, participants also self‐reported any self‐harming behaviours over the prior 12 months (see Table S3 for further detail).

#### 2.2.4 Confounders

A causal diagram was developed a priori to guide the selection of confounders (see Figure S4). These are listed in the ‘Statistical Analysis’ section below, and details on their measurement are provided in Table S3.

### 2.3 Statistical Analysis

Demographic characteristics, mental health problems and SMU were described for the retained participants (*n* = 1195) as frequencies and percentages. Data were structured longitudinally such that, for each wave, confounders were measured at the preceding wave (t–1), SMU at wave t and mental health outcomes at the subsequent wave (*t* + 1). Within this framework, the primary effect of interest was the mean effect of SMU at any given wave (from waves 4 to 10) on mental health problems at the subsequent wave, expressed on risk ratio (RR) and risk difference (RD) scales. This was obtained as the mean of RRs or RDs averaged across all consecutive wave pairs (e.g., SMU at wave 4 on mental health at wave 5, SMU at wave 5 on mental health at wave 6, and so on). The RD scale was examined because it is more appropriate for estimating the potential public health impact of policies aimed at reducing SMU during adolescence. Specifically, RD estimates allow us to infer the absolute number of adolescents who might experience changes in mental health outcomes if exposed to different levels of SMU resulting from policy interventions. Regression standardisation (g‐computation) was used to adjust for confounding. A logistic regression model was fitted for each mental health outcome, with SMU and relevant confounders (see below) as predictors. Using this model, predicted probabilities of the outcomes were estimated for all individuals under three levels of SMU (< 1, 1–2, > 2 h). Separate models were fitted for each outcome (high depressive symptoms, high anxiety symptoms, poor well‐being, self‐harm). The means of the predicted probabilities were then calculated across individuals for each exposure level. RDs were calculated by contrasting the mean of the predicted probabilities (risks) between exposure levels: > 2 h versus 1–2 h, > 2 h versus < 1 h and 1–2 h versus < 1 h. To account for correlated data structure (each participant contributed a separate row for each wave), generalised estimating equations with a logit link function, exchangeable correlation matrix and robust standard errors were used as the outcome model. Standard errors and 95% confidence intervals (CIs) were estimated using the delta method. In keeping with current advice for best practice that discourages dichotomising evidence for an effect [32], we interpreted study findings by considering the strength of point estimates and range of effect sizes with which our data were compatible under modelling assumptions (i.e., values within the 95% CI).

#### 2.3.1 Confounders

All reported RRs and RDs were adjusted for confounders. Models were adjusted for wave 1 measures of sex (female, male), country of birth (Australia, other) and Socio‐Economic Indexes for Areas category (based on the Index of Relative Socio‐economic Advantage and Disadvantage). In addition, the following confounders were drawn from the wave immediately preceding the exposure: depressive symptoms, anxiety symptoms, poor well‐being, peer victimisation, parental support, sleep duration, physical activity and SMU (e.g., when SMU at wave 5 was the exposure, SMU at wave 4 was included as a confounder). For models of self‐harm, we also adjusted for self‐harm measured at the wave before SMU. In sensitivity analyses, we included an additional confounder that indicated the presence of mental health problems at the same wave as SMU, measured as meeting the threshold for high depressive symptoms, high anxiety symptoms or poor well‐being (or self‐harm for the self‐harm outcome models). This adjustment aimed to account for potential confounding by concurrent mental health status. However, this variable was not included in the primary analysis because mental health at the same wave may act as a mediator rather than a confounder.

Models were also adjusted for age (years), under an assumed linear relationship with the outcome, and age period (early, middle or late adolescence) at the time of SMU. We defined age periods as early adolescence (waves 4 and 5, mean ages 12–13 years), middle adolescence (waves 6 to 8, mean ages 14–16 years) and late adolescence (waves 9 and 10, mean ages 17–18 years). The age distribution across waves is outlined in Figure S5.

Based on a priori decisions, interaction terms between SMU and sex, and between SMU and age period, were included in models to relax the assumption that the effects of SMU were constant across strata of these variables. A sex and age period interaction term was also included for more flexible confounding adjustment. We assumed that the effect of SMU on the outcome was constant across strata of all other covariates.

### 2.4 Missing Data

Of the 1195 participants, 79.1% (945) had missing data for at least one variable from waves 1 to 11 (Table S2). Missing data were handled using multiple imputation by fully conditional specification, stratified by sex [33] with 80 complete datasets imputed. All analysis variables (Table S2) were included in imputation models. Variables were imputed in the same form in which they were included in analytic models. Each imputed dataset was analysed separately, and final estimates and 95% CIs were combined using Rubin's rules.

Multiply imputed data were generated using the mice (Multivariate Imputation by Chained Equations) package [34] in R 4.4.2 [35]. All other analyses were performed in Stata 19.0 [36].

### 2.5 STROBE Statement Checklist

This study is reported according to the Strengthening the Reporting of Observational Studies in Epidemiology (STROBE) Statement for cohort studies (see Supporting Information).

### 2.6 Ethical Considerations

Ethics approval was granted by the Royal Children's Hospital Human Research Ethics Committee (#31089). Permission was granted from the Victorian Department of Education and Early Childhood Development and the Catholic Education Office Melbourne to recruit through their schools, and from individual independent schools. Participants were recruited through active, written, informed parent consent. From wave 11, participants themselves provided written informed consent as all had turned 18 years old.

## 3 Results

**FIGURE 1:**
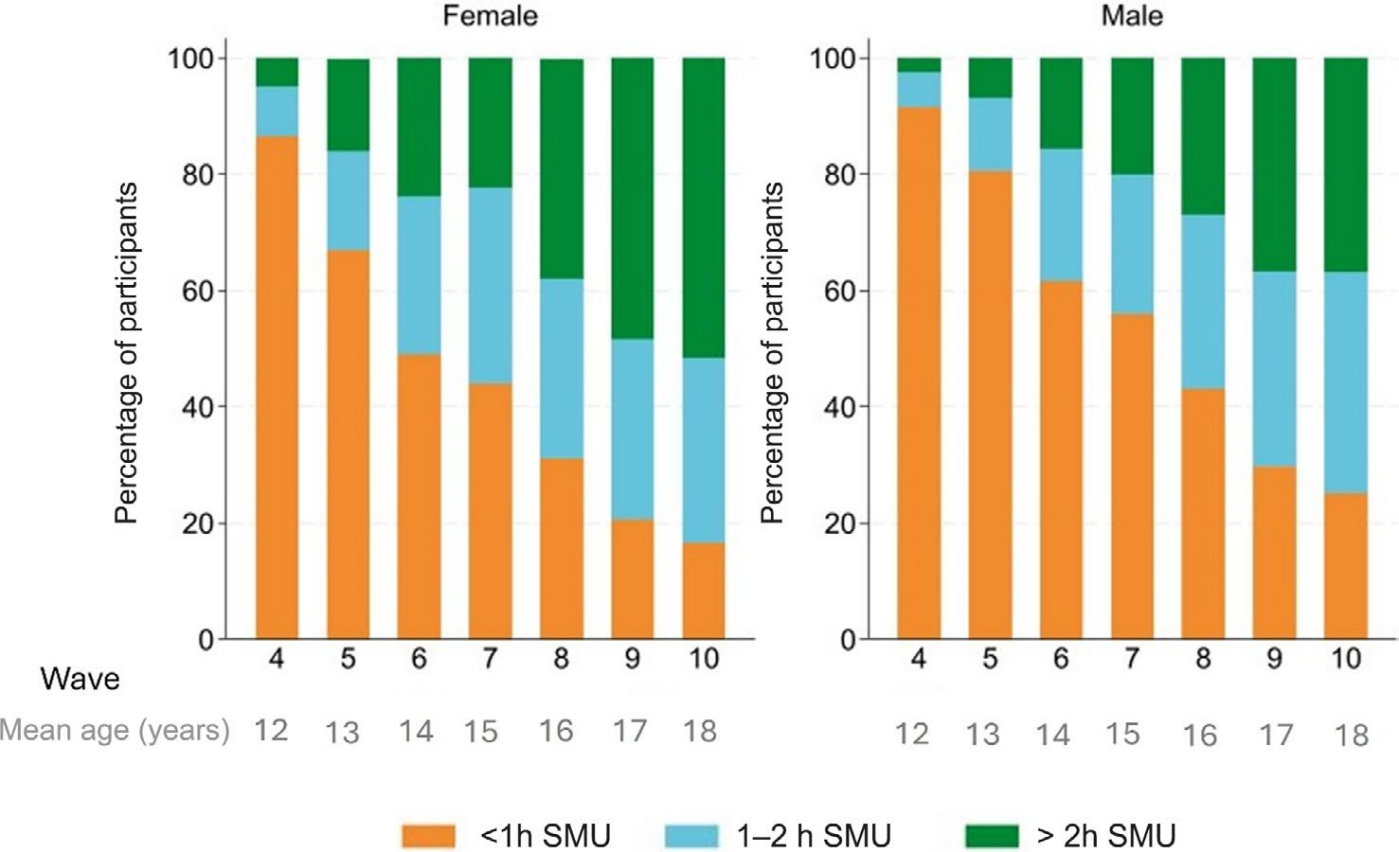
Proportions of social media use (SMU) across waves (spanning 12–18 years of age) among female and male participants (*n* = 1195). At each wave, the standard deviation of the age distribution was 0.4 years.

**FIGURE 2:**
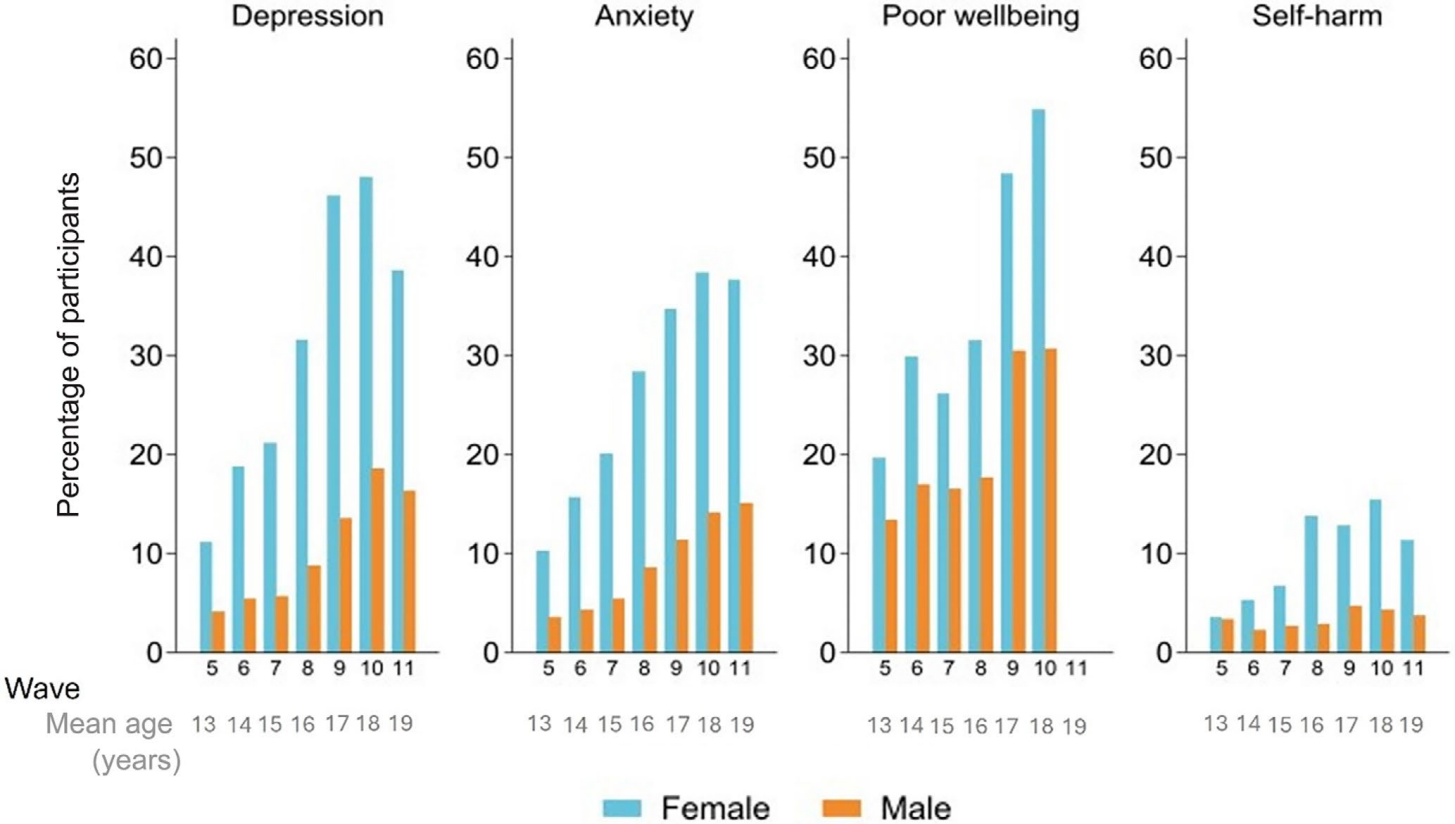
Prevalence of mental health problems (high depressive symptoms, high anxiety symptoms, poor well‐being and self‐harm) across waves (spanning 13–19 years of age) among female and male participants (*n* = 1195). At each wave, the standard deviation of the age distribution was 0.4 years.

Table 1 describes the demographic characteristics of the retained cohort (*n* = 1195). The proportions of adolescents using 1–2 h or > 2 h of social media increased across waves (Figure 1; Table S4). By waves 9 to 10 (ages 17 to 18 years), about 50% of female participants reported > 2 h of use, while most male participants reported 1–2 h or > 2 h of use. The proportion of adolescents reporting mental health problems also tended to increase across waves (Figure 2; Table S5). The highest proportions were reported at wave 10 (mean age of 18 years), when about 40%–50% of female participants and 15%–30% of male participants reported high depressive symptoms, high anxiety symptoms and poor well‐being. Endorsement of self‐harm was lower, although sex differences were still evident, with higher rates in female participants.

**TABLE 1:** Demographic characteristics (wave 1) of the 1195 participants included in the analytic sample.

Participant characteristics	Number (%) or mean [SD]
Male	552 (46)
Age, years	9.0 [0.4]
Aboriginal and Torres Strait Islander status^a^	56 (5)
Born in Australia^b^	1020 (88)
Socioeconomic status (SEIFA)	
1st quintile, most disadvantaged	158 (13)
2nd quintile	103 (9)
3rd quintile	188 (16)
4th quintile	337 (28)
5th quintile, most advantaged	409 (34)

Abbreviations: SD, standard deviation; SEIFA, Socio‐Economic Indexes for Areas.

^a^ *n* = 36 (3%) of the sample had missing data for this variable.

^b^ *n* = 34 (3%) of the sample had missing data for this variable.

### 3.1 Estimated Effects of SMU Across Adolescence

**FIGURE 3:**
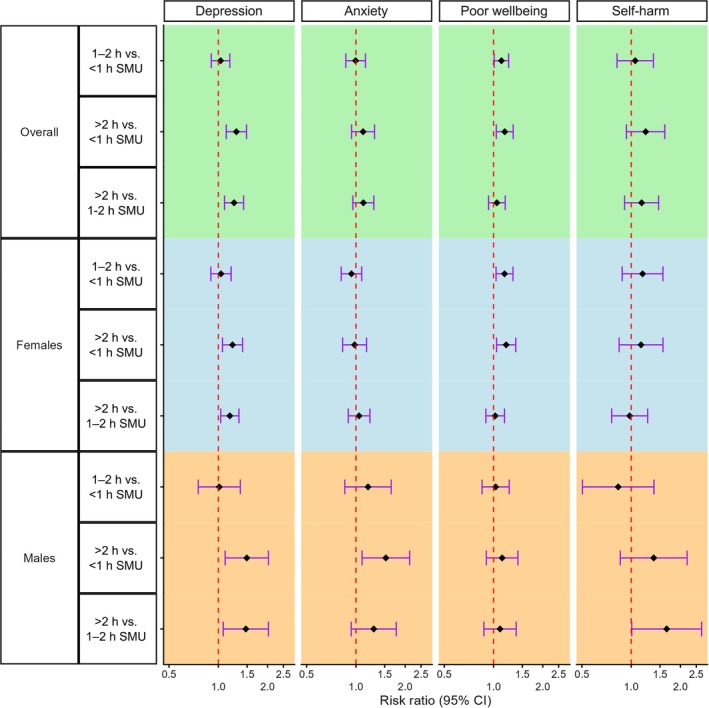
Risk ratios (95% confidence intervals [CIs]) in mental health problems (high depressive symptoms, high anxiety symptoms, poor well‐being and self‐harm) across different levels of social media use (SMU) during adolescence.

**FIGURE 4:**
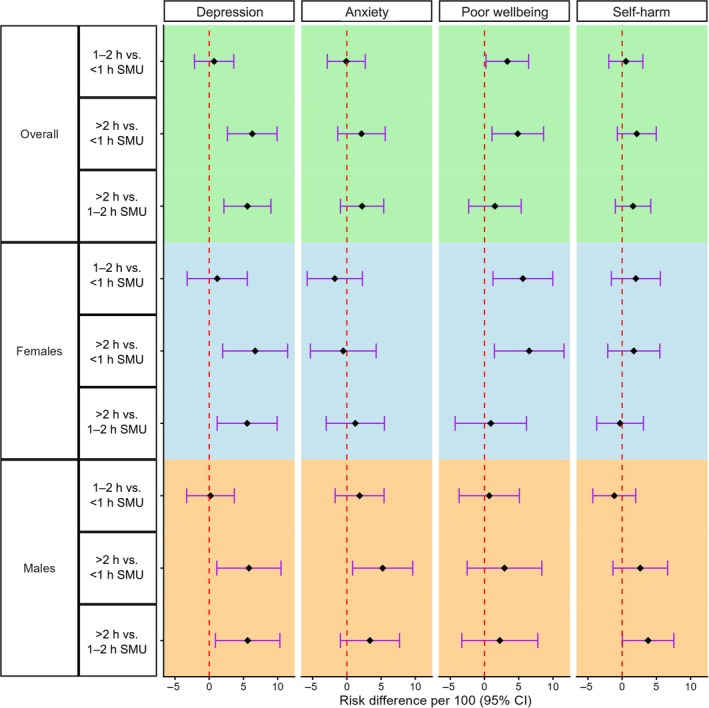
Risk differences (per 100) (95% confidence intervals [CIs]) in mental health problems (high depressive symptoms, high anxiety symptoms, poor well‐being and self‐harm) across different levels of social media use (SMU) during adolescence.

Estimated RRs and RDs (per 100) for the effects of differing levels of SMU on mental health problems 1 year later, across all adolescents and stratified by sex, are illustrated in Figures 3 and 4 (point estimates and 95% CIs are presented in Table S6). Results indicated increased risk for high depressive symptoms in adolescents reporting > 2 h versus < 1 h of SMU (RR, 1.29 [95% CI, 1.12–1.49]; RD, 6.3 [95% CI, 2.7–9.9]) and > 2 h versus 1–2 h of SMU (RR, 1.25 [95% CI, 1.09–1.43]; RD, 5.6 [95% CI, 2.1–9.0]). Sex‐stratified analyses revealed a similar pattern of increased risk in male and female participants. Relative risks were higher in male participants (> 2 h vs. < 1 h: RR, 1.49 [95% CI, 1.10–2.02]; > 2 h vs. 1–2 h: RR, 1.47 [95% CI, 1.07–2.02]) than in female participants (> 2 h vs. < 1 h: RR, 1.22 [95% CI, 1.06 to 1.41]; > 2 h vs. 1–2 h: RR, 1.18 [95% CI, 1.03 to 1.34]), but absolute risk was similar in male participants (> 2 h vs. < 1 h: RD, 5.8 [95% CI, 1.1 to 10.5]; > 2 h vs. 1–2 h: RD, 5.6 [95% CI, 0.9 to 10.3]) and female participants (> 2 h vs. < 1 h: RD, 6.7 [95% CI, 2.0 to 11.4]; > 2 h vs. 1–2 h: RD, 5.5 [95% CI, 1.2 to 9.9]).

Those reporting > 2 h versus < 1 h of SMU also had increased risk of poor well‐being (RR, 1.17 [95% CI, 1.04 to 1.32]; RD, 4.9 [95% CI, 1.1 to 8.6]), as did those with 1–2 h versus < 1 h of SMU (RR, 1.12 [95% CI, 1.01 to 1.24]; RD, 3.3 [95% CI, 0.2 to 6.4]). This increased risk was observed in sex‐stratified analyses for female participants (> 2 h vs. < 1 h: RR, 1.19 [95% CI, 1.04 to 1.37] and RD, 6.5 [95% CI, 1.5 to 11.6]; 1–2 h vs. < 1 h: RR, 1.17 [95% CI, 1.04 to 1.31] and RD, 5.6 [95% CI, 1.2 to 10.0]), but risk estimates in male participants were smaller and 95% CIs were inconclusive.

Overall, estimated RDs and RRs were smaller and 95% CIs were inconclusive for high anxiety symptoms. However, in sex‐stratified analyses, there was increased risk in male participants with > 2 h versus < 1 h of SMU (RR, 1.52 [95% CI, 1.09 to 2.13]; RD, 5.2 [95% CI, 0.8 to 9.6]). Associations were close to null for female participants.

There were no clear patterns of risk for self‐harm across levels of SMU on both RD and RR scales. In sex‐stratified analyses, there was increased risk in male participants with > 2 h versus 1–2 h of SMU (RR [1.65 to 2.69]; RD [3.8 to 7.5]), but associations were close to null for female participants.

### 3.2 Estimated Effects of Social Media by Age Period

**FIGURE 5:**
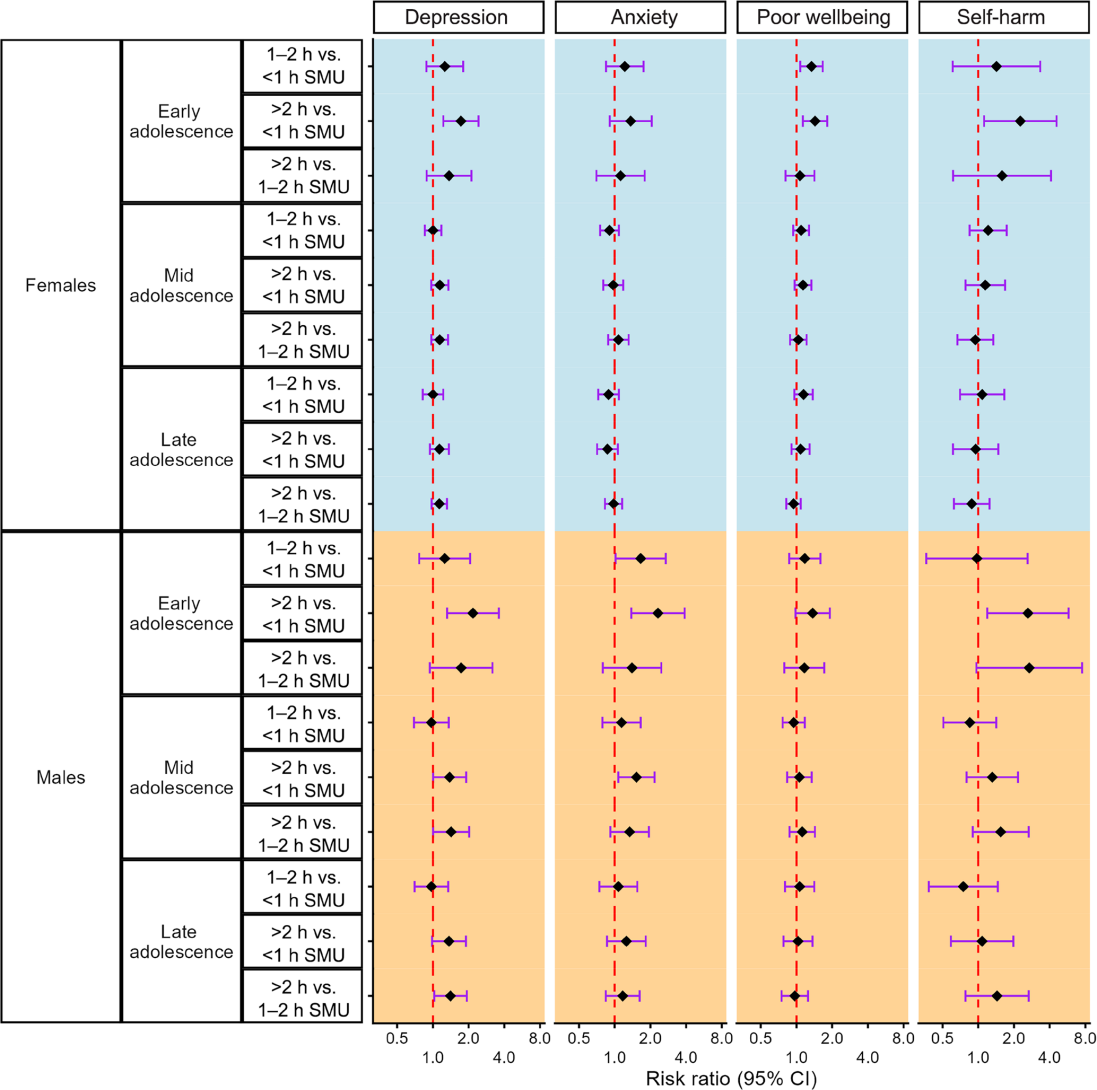
Risk ratios (95% confidence intervals [CIs]) in mental health problems (high depressive symptoms, high anxiety symptoms, poor well‐being and self‐harm) across different levels of social media use (SMU) by age period. Early adolescence: waves 4 and 5, mean ages of 12–13 years. Middle adolescence: waves 6 to 8, mean ages of 14–16 years. Late adolescence: waves 9 and 10, mean ages of 17–18 years.

**FIGURE 6:**
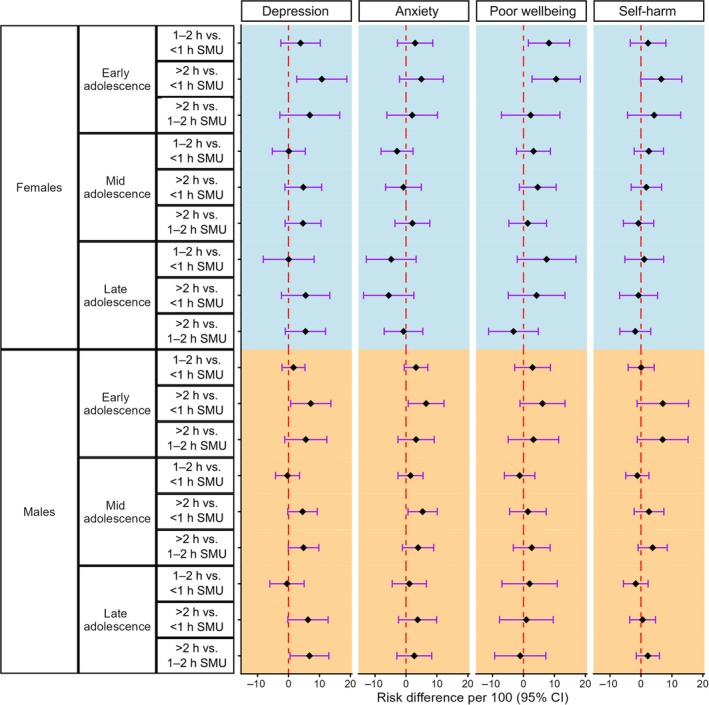
Risk differences (per 100) (95% confidence intervals [CIs]) in mental health problems (high depressive symptoms, high anxiety symptoms, poor well‐being and self‐harm) across different levels of social media use (SMU) by age period. Early adolescence: waves 4 and 5, mean ages of 12–13 years. Middle adolescence: waves 6 to 8, mean ages of 14–16 years. Late adolescence: waves 9 and 10, mean ages of 17–18 years.

Estimated risk for mental health problems by SMU, stratified by age period, are illustrated in Figures 5 and 6 (statistics presented in Table S7). For both female and male participants, general patterns of risk were most pronounced in early adolescence (becoming smaller in later stages of adolescence) and with > 2 h versus < 1 h of SMU.

The largest increased risk of high depressive symptoms in both sexes was for > 2 h versus < 1 h of SMU in early adolescence, with higher absolute risk but lower relative risk for female participants (RR, 1.72 [95% CI, 1.22 to 2.43]; RD, 10.8 [95% CI, 2.7 to 18.9]) compared with male participants (RR, 2.18 [95% CI, 1.32 to 3.59]; RD, 7.2 [95% CI, 0.7 to 13.7]).

For high anxiety symptoms, the largest increased risk was for > 2 h versus < 1 h of SMU in early adolescence in male participants (RR, 2.32 [95% CI, 1.38 to 3.88]; RD, 6.5 [95% CI, 0.7 to 12.3]). A smaller increased risk was also seen for 1–2 h versus < 1 h in early adolescence in male participants (RR, 1.66 [95% CI, 1.02 to 2.69]; RD, 3.2 [95% CI, −0.6 to 7.0]). Associations were close to null for female participants.

For poor well‐being, the largest increased risk was for > 2 h versus < 1 h of SMU in early adolescence, with greater risks observed for female participants both in absolute and relative terms (RR, 1.43 [95% CI, 1.13 to 1.82]; RD, 10.6 [95% CI, 2.8 to 18.4]) compared with male participants (RR, 1.37 [95% CI, 0.98 to 1.91]; RD, 6.2 [95% CI, −1.1 to 13.4]). Among female participants, a smaller but clear increased risk was also seen for 1–2 h versus < 1 h in early adolescence (RR, 1.34 [95% CI, 1.08 to 1.66]; RD, 8.2 [95% CI, 1.6 to 14.9]).

Self‐harm risk was also highest for > 2 h versus < 1 h of SMU in early adolescence, with greater risk in male participants in both relative and absolute terms (female participants: RR, 2.26 [95% CI, 1.12 to 4.57] and RD, 6.6 [95% CI, −0.1 to 13.2]; male participants: RR, 2.62 [95% CI, 1.19 to 5.77] and RD, 7.1 [95% CI, −1.3 to 15.4]).

The patterns of associations from analyses that additionally adjusted for the combined indicator of mental health problems at the concurrent wave as the exposure were similar to those from the primary analyses, in terms of both direction and strength of associations (statistics are presented in Tables S8 and S9).

## 4 Discussion

This study sought to inform debate about the potential effect of SMU on adolescent mental health problems. Using data from CATS, we found that more than 2 h of SMU per day was associated with a small increase in the risk of high depressive symptoms and poor well‐being at the subsequent annual wave across adolescence. Increased risk for all mental health problems was strongest in early adolescence, suggesting this period may be an important period for targeting policies to mitigate the harmful effects of SMU.

Across adolescents aged 12–18 years, our findings suggest that compared with those who used social media less than 1 h/day, there were six additional individuals with high depressive symptoms at the subsequent annual wave for every 100 adolescents who used social media more than 2 h/day. Moreover, we identified similar levels of risk for poor well‐being, but in female adolescents only. These results broadly align with several reviews concluding that the negative effects of social media on mental health problems are small across all adolescents [5, 6], but also with many studies suggesting that effects for well‐being are greater for female relative to male adolescents [22, 24, 37, 38]. As most prior studies have used correlation‐based analyses rather than risk estimates, direct comparisons are challenging. Our findings also provide some support for increased risk for high anxiety symptoms and self‐harm in male adolescents in contrast to the few existing longitudinal studies that have reported null effects for self‐harm [39, 40].

We identified the greatest risk associated with SMU was in early adolescence (12–13 years) for both female and male adolescents. This equated to around 11 additional female adolescents with high depressive symptoms and poor well‐being, and seven additional male adolescents with high depressive symptoms and high anxiety symptoms, for every 100 with more than 2 h SMU per day compared with less than 1 h of use. In some instances, high levels of use were also associated with increased risk in middle and late adolescence, but the estimated effects were about half the size of those for early adolescence. Moreover, even a lesser 1–2 h of social media per day was associated with increased risk of poor well‐being in female adolescents and high anxiety symptoms in male adolescents during early adolescence—an association not evident in later periods. Finally, high levels of SMU also increased risk for self‐harm in early adolescence but not in later periods for both male and female adolescents. These findings collectively provide some support for heightened mental health risks associated with SMU for young adolescents, and suggest that regulation of social media in early adolescence may have the largest public health impacts compared to later age periods.

Our findings broadly align with those from Orben and colleagues [21], who showed that female adolescents aged 11–13 years who increased their SMU reported poorer life satisfaction 1 year later (within‐subject associations), with no such associations observed in 14–18‐year‐olds. While Orben and colleagues reported similar associations between SMU and life satisfaction for male adolescents aged 14–15 years, we observed that the greatest mental health risk for male adolescents was also in early adolescence. It remains to be determined whether this inconsistency reflects our focus on between‐subject effects (i.e., comparing individuals with > 2 h vs. < 1 h of SMU) or other methodological differences. Of note, several prior longitudinal studies have failed to identify age‐related differences when considering social media effects across male and female adolescents [16, 17, 18, 25, 41], underscoring the need to consider how sex and age may jointly influence the potential benefits from social media regulations.

Many young people first use social media and learn to navigate its complexities during early adolescence. As certain cognitive and regulatory capacities are less developed at this time [42], this may make it difficult to manage emotionally intense experiences on social media, such as idealised social comparisons (e.g., of physical appearance), bullying and exposure to risky behaviours [43]. Moreover, peer approval and body image become highly salient (particularly for female adolescents) during early adolescence, leading them to engage with more appearance‐focussed content and associated social comparisons. The combination of increased social media engagement, heightened social sensitivity and immature regulatory capacities may contribute to increased risk during early adolescence. In the context of sex differences in the prevalence of mental health problems during early adolescence [44, 45], this risk also results in a higher absolute number of female adolescents affected by SMU relative to male adolescents.

## 5 Limitations

The strengths of the current study provide a strong foundation for understanding causal inference at the population level. The Child to Adult Transition Study is one of few cohorts to annually track the same individuals over the course of adolescence. Findings may be broadly applicable to adolescents in other high‐income Western countries that have parallel high levels of social media engagement and adolescent mental health problems. We examined adolescents' SMU from 2014 to 2021, yet social media platforms, features and patterns of use changed considerably during this period, and effects on mental health may also have changed. To maintain comparability of exposure across waves in this long‐running cohort, we measured overall duration of SMU rather than platform‐specific behaviours. However, duration is a relatively coarse indicator and does not capture differences in the type or context of engagement, such as “active” versus “passive” engagement or exposure to specific types of content or experiences (e.g., cyberbullying). Adolescents also tend to under‐report their SMU [46], reflecting the need for more objective assessments in future. Moreover, because both SMU and mental health were self‐reported, our estimates may also be affected by measurement bias arising from dependent measurement error. We were also limited to categorising “high” levels of SMU as more than 2 h per day due to small levels of endorsement in early adolescence. However, there may be important risk differences in durations above 2 h per day for older adolescents. Similarly, our study did not distinguish between no use and low use (up to 1 h) due to low numbers in these categories (particularly in later adolescence), which is an important limitation of the conclusions we can draw about age‐based restrictions.

Although we used a lagged exposure–outcome design, adjusted for prior mental health and a range of relevant confounders informed by a causal diagram, and conducted sensitivity analyses adjusting for contemporaneous mental health, alternative explanations, including residual confounding, reverse causation and shared underlying vulnerabilities that may influence both SMU and mental health, cannot be fully excluded. We used multiple imputation to address missing data, including missingness due to attrition. This approach can be expected to yield estimates with negligible bias provided that, conditional on the variables included in the imputation model, the outcome does not cause its own missingness [47, 48]. This assumption may have been violated in our study, which could introduce selection bias. Finally, the late adolescent waves of data collection occurred during the coronavirus disease 2019 (COVID‐19) pandemic, which may have influenced adolescents' SMU and mental health. However, the largest associations observed in this study occurred during early adolescence, well before the pandemic period. It is also likely that the effects of social media differ across other subgroups of adolescents, such as those with pre‐existing mental health problems or those from marginalised communities, which represents an important avenue for future research.

## 6 Conclusions

In summary, more than 2 h of SMU per day was associated with a small increased risk for high depressive symptoms in adolescents 1 year later, and a similar increased risk for poor well‐being in female adolescents, as well as high anxiety symptoms and self‐harm in male adolescents. Increased risk differences for all mental health problems were most pronounced during early adolescence, when even 1–2 h of SMU increased the risk of some mental health problems. Our findings suggest that in terms of the absolute number of individuals impacted, early adolescence may represent a key developmental period in which policies aimed at reducing high levels of SMU could have the greatest potential population‐level impact. Such policies would also need to be complemented by developmentally sensitive supports such as parental guidance, school‐based media literacy and well‐being programs, and platform‐level safeguards.

## Author Contributions

**Nandita Vijayakumar:** conceptualisation, methodology, original draft, funding acquisition. **S. Ghazaleh Dashti**, conceptualisation, methodology, formal analysis, original draft, funding acquisition. **Louise Canterford:** methodology, data curation, formal analysis, original draft. **Susan Ellul:** methodology, formal analysis, original draft. **Anthony J. A. Parissi:** methodology, formal analysis, writing – review and editing. **Anne‐Lise Goddings:** writing – review and editing. **Russell M. Viner:** writing – review and editing, funding acquisition. **Paul Moran:** data curation, review and editing. **Rohan Borschmann:** data curation, writing – review and editing, funding acquisition. **Lisa K. Mundy:** writing – review and editing, funding acquisition. **Ellie M. Robson:** writing – review and editing. **Susan M. Sawyer:** conceptualisation, methodology, writing – review and editing, funding acquisition.

## Funding

This study was funded by the National Health and Medical Research Council (NHMRC) of Australia, GNT 1010018 (2011‐15) and GNT 1122189 (2017–20), as well as a grant from the Royal Children's Hospital Foundation. It was also supported by the Melbourne Children's LifeCourse Initiative, which has received funding from the Victorian Government's Department of Health and Human Services, Victor Chiodo Foundation and Morgan Stanley. The following authors are supported by National Health and Medical Research Council Investigator Grants: SMS (GNT 1196999), SGD (202171) and RB (GNT 2008073). RB receives salary support from the Better Health & Care Hub at King's College London, UK. Permission was granted from the Victorian Department of Education and Early Childhood Development and the Catholic Education Office Melbourne to recruit through schools. The funding sources had no role in the study design; in the collection, analysis and interpretation of data; in the writing of the report; and in the decision to submit the paper for publication.

## Disclosure

Not commissioned; externally peer reviewed.

## Conflicts of Interest

The authors declare no conflicts of interest.

## Supporting information


**Figure S1:** Flowchart of CATS participants (*N* = 1239) from recruitment onwards.
**Table S1:** Wave 1 demographic characteristics of the adolescents included in the current study (*N* = 1195) compared to the full Childhood to Adult Transition Study cohort (*N* = 1239).
**Table S2:** Frequency and percent of missing data for all variables, overall and by sex.
**Figure S2:** Distribution of social media use (on a normal school day) at each of waves 4–10. At each wave, the standard deviation of the age distribution was 0.4 years.
**Figure S3:** Proportion of sample using popular social media sites from waves 4 to 10. At each wave, the standard deviation of the age distribution was 0.4 years.
**Table S3:** Information about how depressive and anxiety symptoms were measured at waves 3–11 and description of confounders included in the analytic models. All measures were self‐report apart from SES, which was based on home postcode by parent‐report.
**Figure S4:** Causal diagram that guided confounder selection.
**Figure S5:** Distribution of age at each of waves 3–11.
**Table S4:** Number (percentage) of study participants (*N* = 1195) in each social media use category (on a typical school day), at each of waves 4–10, by sex.
**Table S5:** Number (percentage) of study participants (*N* = 1195) reporting high depressive symptoms, high anxiety symptoms, and poor general well‐being, at each of waves 5–11, by sex.
**Table S6:** Estimated risk ratios (95% CI) and risk differences (95% CI) of social media use (on a typical week day) on future mental health (high depressive symptoms, high anxiety symptoms, poor well‐being, self‐harm), over adolescence, overall and by sex.
**Table S7:** Estimated risk ratios (95% CI) and risk differences (95% CI) of social media use (on a typical week day) on future mental health (high depressive symptoms, high anxiety symptoms, poor well‐being, self‐harm), by period of adolescence and by sex.
**Table S8:** Estimated risk ratios (95% CI) and risk differences (95% CI) of social media use (on a typical week day) on future mental health (high depressive symptoms, high anxiety symptoms, poor well‐being, self‐harm), over adolescence, overall and by sex.
**Table S9:** Estimated risk ratios (95% CI) and risk differences (95% CI) of social media use (on a typical week day) on future mental health (high depressive symptoms, high anxiety symptoms, poor well‐being, self‐harm), by period of adolescence and by sex.

## Data Availability

The de‐identified data we analysed are not publicly available. Details of this data source can be found at https://lifecourse.melbournechildrens.com/cohorts/cats/. Requests to access data can also be submitted here.
