# Supplementary material for: The Effects of Social Media on Adolescent Mental Health: Findings From a Population‐Based Cohort Study in Australia
Source: Med J Aust. 2026 Jun 11;224(6):e70220. doi: 10.5694/mja2.70220 (PMC13256062; doi:10.5694/mja2.70220)
Supplement: Supplementary file 1 — Figure S1: Flowchart of CATS participants (N = 1239) from recruitment onwards. Table S1: Wave 1 demographic characteristics of the adolescents included in the current study (N = 1195) compared to the full Childhood to Adult Transition Study cohort (N = 1239). Table S2: Frequency and percent of missing data for all variables, overall and by sex. Figure S2: Distribution of social media use (on a normal school day) at each of waves 4–10. At each wave, the standard deviation of the age distribution was 0.4 years. Figure S3: Proportion of sample using popular social media sites from waves 4 to 10. At each wave, the standard deviation of the age distribution was 0.4 years. Table S3: Information about how depressive and anxiety symptoms were measured at waves 3–11 and description of confounders included in the analytic models. All measures were self‐report apart from SES, which was based on home postcode by parent‐report. Figure S4: Causal diagram that guided confounder selection. Figure S5: Distribution of age at each of waves 3–11. Table S4: Number (percentage) of study participants (N = 1195) in each social media use category (on a typical school day), at each of waves 4–10, by sex. Table S5: Number (percentage) of study participants (N = 1195) reporting high depressive symptoms, high anxiety symptoms, and poor general well‐being, at each of waves 5–11, by sex. Table S6: Estimated risk ratios (95% CI) and risk differences (95% CI) of social media use (on a typical week day) on future mental health (high depressive symptoms, high anxiety symptoms, poor well‐being, self‐harm), over adolescence, overall and by sex. Table S7: Estimated risk ratios (95% CI) and risk differences (95% CI) of social media use (on a typical week day) on future mental health (high depressive symptoms, high anxiety symptoms, poor well‐being, self‐harm), by period of adolescence and by sex. Table S8: Estimated risk ratios (95% CI) and risk differences (95% CI) of social media use (on a typical week [file MJA2-224-0-s001.pdf]

Supporting Information

Supplementary material

This appendix was part of the submitted manuscript and has been peer reviewed.
It is posted as supplied by the authors.

Appendix to: N. Vijayakumar, S. G. Dashti, L Canterford, et al. The Effects of Social Media on Adolescent Mental Health: Findings from a Population-Based Cohort Study in Australia. *Med J Aust* 2026; doi: 10.5694/mja2.70220.

Supplementary Material for 'The effects of social media on adolescent mental health: Findings from a population-based cohort study in Australia'

Table of contents

Flowchart of participants from recruitment onwards 3

CATS cohort description 4

Summary of missing data 5

Distribution of social media use 8

Detailed description of variables included in analyses 9

Causal diagram that guided confounder selection 11

Distribution of age 12

Distribution of social media use 13

Distribution of outcome measures 14

Estimated risk ratios and risk differences of social media use on future mental health 16

Sensitivity analysis: Estimated risk differences and risk ratios of social media use on future mental health, after inclusion of a measure of the presence of any mental health problems measured at same wave as exposure 19

STROBE Statement—checklist of items that should be included in reports of observational studies 22

Supplementary Material Reference List 27

Flowchart of participants from recruitment onwards

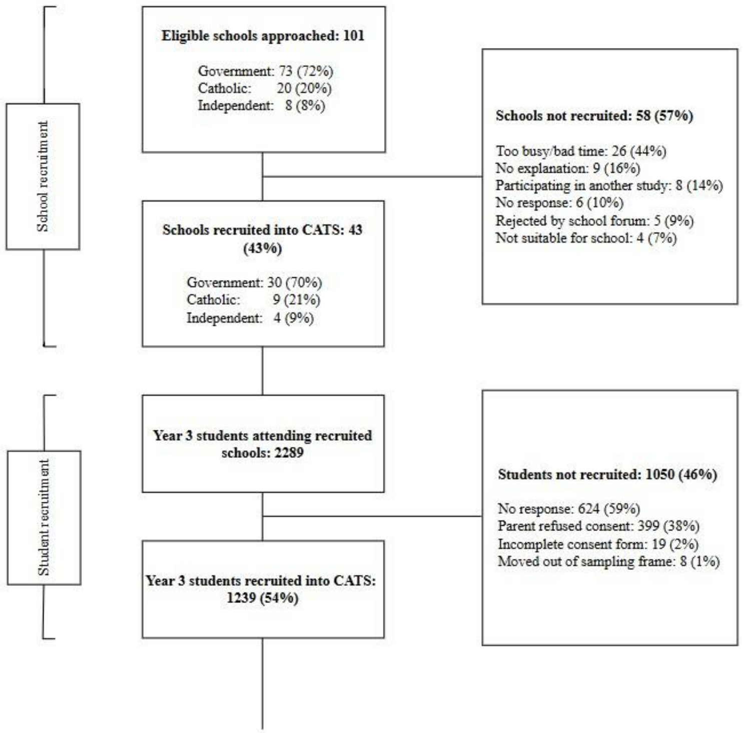

Wave	1	2	3	4	5	6	7	8	9	10	11
Year	2012	2013	2014	2015	2016	2017	2018	2019	2020	2021	2022
School Year	3	4	5	6	7	8	9	10	11	12	-
Mean (SD) age (years)	9.0 (0.4)	9.9 (0.4)	10.9 (0.4)	11.9 (0.4)	13.0 (0.4)	13.8 (0.4)	14.8 (0.4)	15.8 (0.4)	16.9 (0.4)	17.8 (0.4)	18.9 (0.4)
N	1,194	1,158	1,119	1,068	996	1,001	900	856	848	835	832
Response rate# (%)	96.4	93.5	90.3	86.2	80.4	80.8	72.6	69.1	68.4	67.4	67.1

Based on the number of completed young person surveys as a proportion of the recruited sample (n = 1,239). SD = Standard Deviation

FIGURE S1: Flowchart of CATS participants (N = 1,239) from recruitment onwards.

CATS cohort description

The CATS cohort (N = 1,239) had a similar socioeconomic position (based on the Australian Bureau of Statistics Socio-Economic Index for Areas (SEIFA) 2011: Index of Relative Socio-economic Advantage and Disadvantage; mean = 1012, standard deviation [SD] = 67) as metropolitan Melbourne at baseline (Australian Bureau of Statistics, 2011). While reflective of the Melbourne population at baseline, our cohort had an over-representation of minoritised communities (e.g., Aboriginal and Torres Strait Islanders) and under-representation of socioeconomically disadvantaged communities, which restricts the generalisability of our findings to populations with greater diversity. Demographic characteristics of the 1,195 participants (552 male participants) used in the current study were similar to the full cohort at wave 1 (see Supplementary Table S1).

TABLE S1: Wave 1 demographic characteristics of the adolescents included in the current study (N=1,195) compared to the full Childhood to Adult Transition Study cohort (N=1,239).

Measure	Current sample	Full cohort
	N (%) / Mean (SD)	N (%) / Mean (SD)
**Participant characteristics**		
Male; N (%)	552 (46)	572 (46)
Age in years; M (SD)	9.0 (0.4)	9.0 (0.4)
Aboriginal and Torres Strait Islander status; N (%) ‡	56 (5)	58 (5)
Born in Australia; N (%) ^	1020 (88)	1055 (88)
**Socioeconomic status (SEIFA)**; N (%)		
1st quintile (most disadvantaged)	158 (13)	167 (14)
2nd quintile	103 (9)	109 (9)
3rd quintile	188 (16)	194 (16)
4th quintile	337 (28)	346 (28)
5th quintile (most advantaged)	409 (34)	423 (34)

; SEIFA = Socio-Economic Indexes for Areas.

‡ Measured by parent report (Item: 'Is your child Aboriginal or Torres Strait Islander?'; Response options: No, Yes); n = 36 (3%) of current sample cohort had missing data on this measure; 39 (3%) of recruited cohort had missing data on this measure.

^ 34 (3%) of current sample cohort had missing data on this measure; 37 (3%) of recruited cohort had missing data on this measure.

Summary of missing data

TABLE S2: Frequency and percent of missing data for all variables, overall and by sex.

Variable	Overall		Females		Males	
	n	%‡	n	%^	n	%#
Wave 3 depressive symptoms	127	10.6	68	10.6	59	10.7
Wave 4 depressive symptoms	144	12.1	88	13.7	56	10.1
Wave 5 depressive symptoms	263	22.0	147	22.9	116	21.0
Wave 6 depressive symptoms	219	18.3	127	19.8	92	16.7
Wave 7 depressive symptoms	319	26.7	174	27.1	145	26.3
Wave 8 depressive symptoms	362	30.3	187	29.1	175	31.7
Wave 9 depressive symptoms	365	30.5	177	27.5	188	34.1
Wave 10 depressive symptoms	382	32.0	185	28.8	197	35.7
Wave 11 depressive symptoms	376	31.5	174	27.1	202	36.6
Wave 3 anxiety symptoms	116	9.7	62	9.6	54	9.8
Wave 4 anxiety symptoms	153	12.8	88	13.7	65	11.8
Wave 5 anxiety symptoms	214	17.9	121	18.8	93	16.8
Wave 6 anxiety symptoms	207	17.3	121	18.8	86	15.6
Wave 7 anxiety symptoms	312	26.1	171	26.6	141	25.5
Wave 8 anxiety symptoms	351	29.4	184	28.6	167	30.3
Wave 9 anxiety symptoms	361	30.2	176	27.4	185	33.5
Wave 10 anxiety symptoms	381	31.9	183	28.5	198	35.9
Wave 11 anxiety symptoms	373	31.2	172	26.7	201	36.4
Wave 3 poor general wellbeing	79	6.6	43	6.7	36	6.5
Wave 4 poor general wellbeing	128	10.7	81	12.6	47	8.5
Wave 5 poor general wellbeing	209	17.5	118	18.4	91	16.5
Wave 6 poor general wellbeing	200	16.7	118	18.4	82	14.9
Wave 7 poor general wellbeing	308	25.8	168	26.1	140	25.4
Wave 8 poor general wellbeing	347	29.0	179	27.8	168	30.4
Wave 9 poor general wellbeing	358	30.0	173	26.9	185	33.5
Wave 10 poor general wellbeing	375	31.4	181	28.1	194	35.1
Wave 4 self-harm	136	11.4	87	13.5	49	8.9
Wave 5 self-harm	228	19.1	133	20.7	95	17.2
Wave 6 self-harm	231	19.3	137	21.3	94	17.0
Wave 7 self-harm	320	26.8	178	27.7	142	25.7
Wave 8 self-harm	367	30.7	193	30.0	174	31.5
Wave 9 self-harm	377	31.5	183	28.5	194	35.1
Wave 10 self-harm	394	33.0	194	30.2	200	36.2
Wave 11 self-harm	387	32.4	184	28.6	203	36.8
Wave 3 social media use (weekday)	342	28.6	167	26.0	175	31.7
Wave 4 social media use (weekday)	137	11.5	84	13.1	53	9.6
Wave 5 social media use (weekday)	217	18.2	121	18.8	96	17.4
Wave 6 social media use (weekday)	204	17.1	122	19.0	82	14.9
Wave 7 social media use (weekday)	315	26.4	171	26.6	144	26.1
Wave 8 social media use (weekday)	353	29.5	182	28.3	171	31.0

Wave 9 social media use (weekday)	360	30.1	174	27.1	186	33.7
Wave 10 social media use (weekday)	377	31.5	181	28.1	196	35.5
Wave 3 peer victimisation	99	8.3	57	8.9	42	7.6
Wave 4 peer victimisation	134	11.2	85	13.2	49	8.9
Wave 5 peer victimisation	230	19.2	129	20.1	101	18.3
Wave 6 peer victimisation	207	17.3	122	19.0	85	15.4
Wave 7 peer victimisation	318	26.6	172	26.7	146	26.4
Wave 8 peer victimisation	354	29.6	181	28.1	173	31.3
Wave 9 peer victimisation	365	30.5	177	27.5	188	34.1
Wave 3 parental support	91	7.6	53	8.2	38	6.9
Wave 4 parental support	140	11.7	88	13.7	52	9.4
Wave 5 parental support	222	18.6	129	20.1	93	16.8
Wave 6 parental support	218	18.2	130	20.2	88	15.9
Wave 7 parental support	326	27.3	183	28.5	143	25.9
Wave 8 parental support	366	30.6	187	29.1	179	32.4
Wave 9 parental support	379	31.7	190	29.5	189	34.2
Wave 3 sleep duration	171	14.3	85	13.2	86	15.6
Wave 4 sleep duration	134	11.2	82	12.8	52	9.4
Wave 5 sleep duration	218	18.2	123	19.1	95	17.2
Wave 6 sleep duration	216	18.1	123	19.1	93	16.9
Wave 7 sleep duration	310	25.9	171	26.6	139	25.2
Wave 8 sleep duration	362	30.3	183	28.5	179	32.4
Wave 9 sleep duration	376	31.5	182	28.3	194	35.1
Wave 3 physical activity	98	8.2	54	8.4	44	8.0
Wave 4 physical activity	176	14.7	107	16.6	69	12.5
Wave 5 physical activity	247	20.7	131	20.4	116	21.0
Wave 6 physical activity	215	18.0	123	19.1	92	16.7
Wave 7 physical activity	313	26.2	173	26.9	140	25.4
Wave 8 physical activity	356	29.8	184	28.6	172	31.2
Wave 9 physical activity	355	29.7	173	26.9	182	33.0
Wave 4 any mental health problems	161	13.5	93	14.5	68	12.3
Wave 5 any mental health problems	259	21.7	142	22.1	117	21.2
Wave 6 any mental health problems	219	18.3	127	19.8	92	16.7
Wave 7 any mental health problems	320	26.8	174	27.1	146	26.4
Wave 8 any mental health problems	362	30.3	188	29.2	174	31.5
Wave 9 any mental health problems	363	30.4	175	27.2	188	34.1
Wave 10 any mental health problems	382	32.0	182	28.3	200	36.2
Wave 5 any mental health problems/self-harm	269	22.5	149	23.2	120	21.7
Wave 6 any mental health problems/self-harm	236	19.7	138	21.5	98	17.8
Wave 7 any mental health problems/self-harm	326	27.3	178	27.7	148	26.8
Wave 8 any mental health problems/self-harm	370	31.0	192	29.9	178	32.2
Wave 9 any mental health problems/self-harm	369	30.9	178	27.7	191	34.6
Wave 10 any mental health problems/self-harm	384	32.1	184	28.6	200	36.2
Wave 4 age at completion of survey	128	10.7	81	12.6	47	8.5

Wave 5 age at completion of survey	202	16.9	116	18.0	86	15.6
Wave 6 age at completion of survey	198	16.6	117	18.2	81	14.7
Wave 7 age at completion of survey	296	24.8	165	25.7	131	23.7
Wave 8 age at completion of survey	339	28.4	175	27.2	164	29.7
Wave 9 age at completion of survey	348	29.1	167	26.0	181	32.8
Wave 10 age at completion of survey	362	30.3	174	27.1	188	34.1
Country of birth	34	2.8	15	2.3	19	3.4
Sex assigned at birth	0	0	0	0	0	0
SEIFA IRSAD	0	0	0	0	0	0
Missing data in at least one variable	945	79.1	506	78.7	439	79.5

SEIFA IRSAD – Socio-Economic Index for Areas - Index of Relative Socio-Economic Advantage and Disadvantage.

‡ Denominator: N = 1,195

^ Denominator: N = 643

Denominator: N = 552

Distribution of social media use

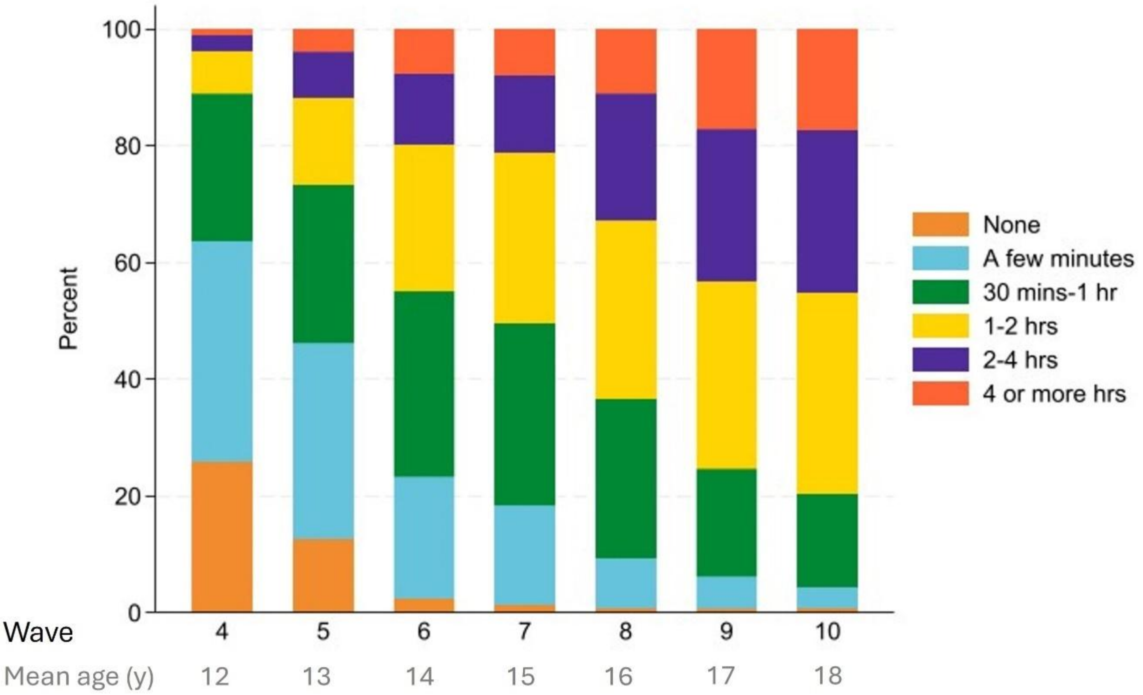

FIGURE S2: Distribution of social media use (on a normal school day) at each of waves 4 to 10. At each wave, the standard deviation of the age distribution was 0.4 years.

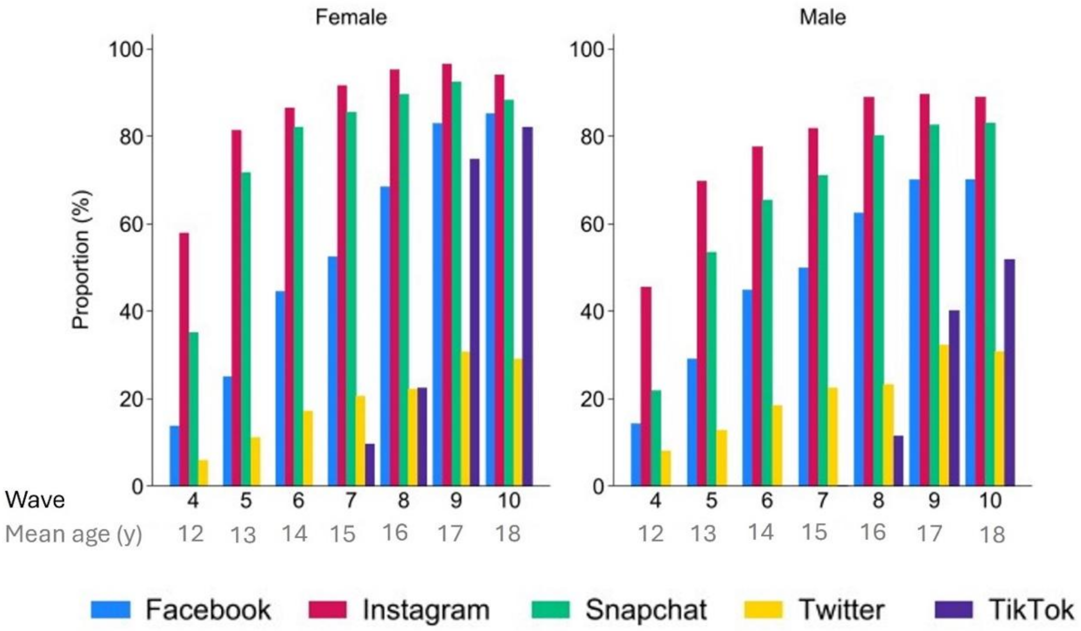

FIGURE S3: Proportion of sample using popular social media sites from waves 4 to 10. At each wave, the standard deviation of the age distribution was 0.4 years.

Detailed description of variables included in analyses

TABLE S3: Information about how depressive and anxiety symptoms were measured at waves 3-11 and description of confounders included in the analytic models. All measures were self-report apart from SES, which was based on home postcode by parent-report

Construct	Measure	Waves	Additional information
Socioeconomic status	Australian Bureau of Statistics Socio-Economic Index for Areas (SEIFA) 2011: Index of Relative Socio-economic Advantage and Disadvantage (IRSAD) (based on home postcode) (population mean = 1000, SD = 100) (Australian Bureau of Statistics)	1	Higher scores indicate a relative lack of disadvantage and greater advantage. Lower scores indicate a relatively greater disadvantage and lack of advantage. Scores were collapsed into a binary variable (more disadvantaged versus more advantaged).
Peer victimisation	Selected items from the Gatehouse Bullying Scale (Bond, Wolfe, Tollit, Butler, & Patton, 2007)	3 to 9	Occurrence ('yes' or 'no') and frequency (if responded 'yes'; 'less than once a week', 'about once a week', or 'most days') of bullying in the past month was measured: physical and verbal bullying (all waves), relational bullying (from wave 2 onwards), and cyberbullying (from wave 3 onwards). At each wave, a binary variable was generated to define the presence of frequent bullying. Those responding either 'about once a week' or 'most days' to at least one form of bullying were classified as 'frequently bullied'.
Parent support	Study specific	3 to 9	At each wave, young people were asked the following questions: "Do you talk about your feelings with your mum?" and "Do you talk about your feelings with you dad?" If participants responded "yes" to either question, they were coded as having parental support.
Sleep duration		3 to 9	At each wave, young people were asked the following questions: "During the past 4 weeks, at what time have you usually turned out the light and gone to sleep on school days (i.e. Monday to Thursday night)?" and "During the past 4 weeks, at what time have you usually woken up in the morning on school days (i.e. Monday to Friday mornings)?". Sleep duration was calculated by subtracting time to sleep from awake time.
Physical activity		3 to 9	At each wave, young people were asked: "Over the last 7 days, on how many days were you physically active for a total of at least 60 minutes (1 hour) per day?".
Depressive symptoms	Short Mood and Feelings Questionnaire (SMFQ)	3 to 11	Two items ('I felt miserable and unhappy' and 'I didn't enjoy anything at all') from the SMFQ were used to assess depressive symptoms at wave 2. Items were scored on a 5-point Likert scale (0 (never) to 4 (almost always)) and responses were then recoded to a 3-point scale (0 (not true), 1 (sometimes true), and 2 (true)) to match the original SMFQ scoring. The total score (ranging from 0-4) was then dichotomised to define the presence of depressive symptoms using a cut-point of $\geq$2 (Rhew et al. 2010).

			At each of waves 3 to 11, all 13 items of the SMFQ were included in the survey. Items were scored and recoded as per wave 2. The total score (ranging from 0-26) was dichotomised at a threshold of ≥12 to define the presence of high levels of depressive symptoms (Thabrew et al. 2018).
Anxiety symptoms	Spence Children's Anxiety Scale (SCAS)	3 to10	Two items ('I worry about things' and 'I feel afraid') from the SCAS were used to assess anxiety symptoms at wave 2. Items were scored on a 5-point Likert scale, ranging from 0 (never) to 4 (almost always). They were recoded to a 4-point scale (0 (never) to 3 (always)) to match the original SCAS scoring. The total score (ranging from 0-6) was then dichotomised to define the presence of anxiety symptoms using a cut-point of ≥3 (a child scoring ≥3 answered at least 'often' on at least one of the two items).<br><br>At each of waves 3 to 10, all 8 items of the SCAS were included in the survey. Items were scored and recoded as per wave 2. The total score (ranging from 0-24) was dichotomised at a threshold of ≥11 to define the presence of high levels of anxiety symptoms.
	7-item Generalised Anxiety Disorder Assessment (GAD-7).	11	Items were scored on a 4-point scale, ranging from 0 (not at all) to 3 (nearly every day). The total score (ranging from 0-21) was dichotomised at a threshold of ≥10 to define the presence of high levels of anxiety symptoms (Kroenke et al. 2007).
Poor wellbeing	Paediatric Quality of Life General Wellbeing Scale	3 to 10	Three items from the Paediatric Quality of Life General Wellbeing Scale were used to assess wellbeing at wave 2. At each of waves 3 to 10, all 6 items of the scale were included in the survey. Mean scores (ranging from 0-4) were linearly transformed to a scale ranging from 0-100, and then dichotomised at <60 to define poor wellbeing.
Self-harm	Study specific	4 to 10	Participants were asked: "In the past 12 months have you ever hurt yourself on purpose or done anything that might have harmed you or even killed you?" If endorsed, participants were asked to describe what they did. Two mental health clinician investigators independently rated the free-text descriptions using a classification system adapted from a previous large-scale epidemiological study of adolescent self-harm (Moran et al., 2012) to distinguish self-harm from behaviours that did not meet criteria.
Any mental health problems		4 to 10	A combined measure indicating the presence of at least one of the following at the same wave: high depressive symptoms, high anxiety symptoms, and poor wellbeing
Any mental health problems and/or self-harm			A combined measure indicating the presence of at least one of the following at the same wave: high depressive symptoms, high anxiety symptoms, poor wellbeing, and self-harm.

Causal diagram that guided confounder selection

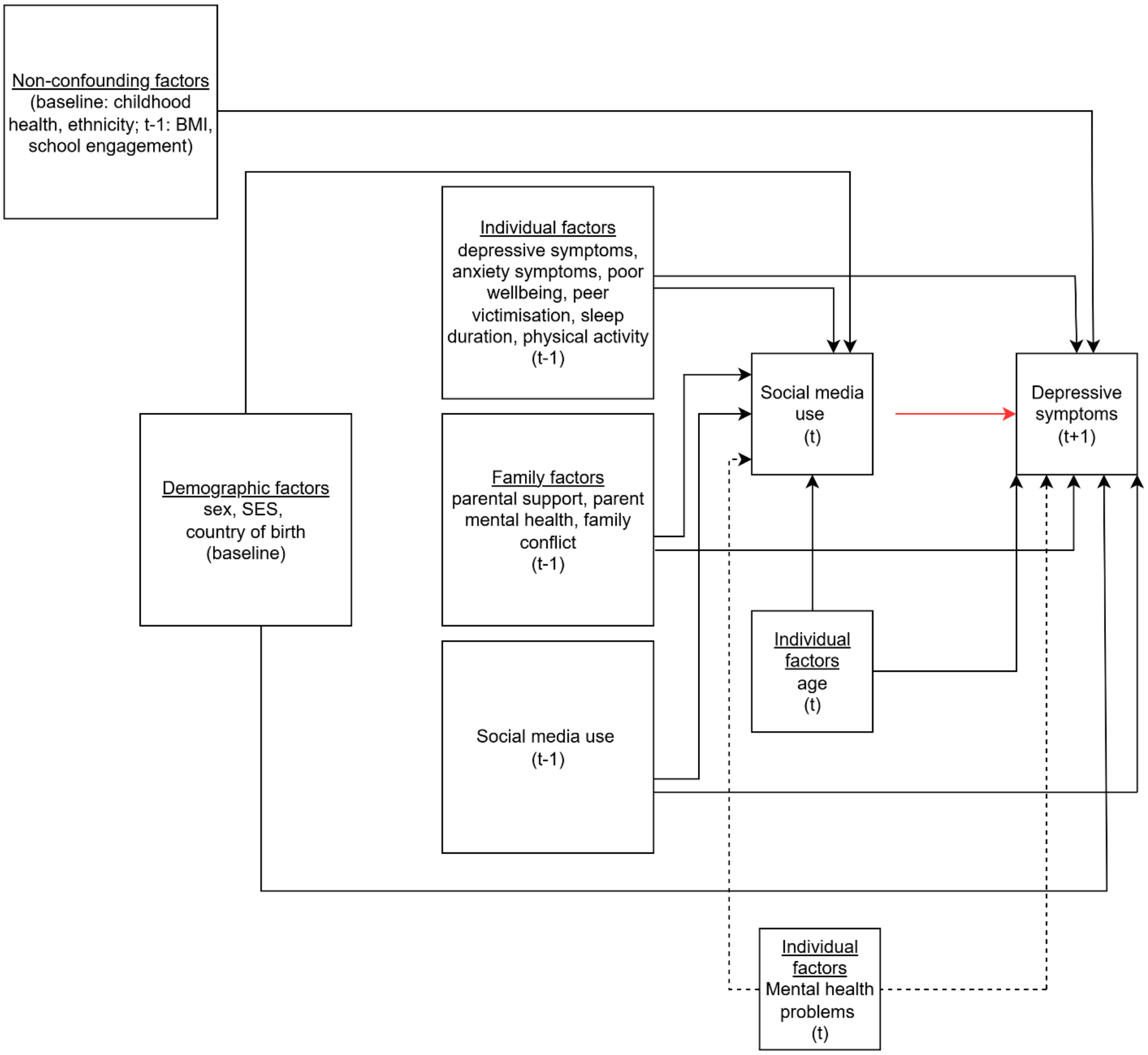

FIGURE S4: Causal diagram that guided confounder selection.

Notes: t = time point / wave

Similar causal diagrams were drawn for each outcome measure: depressive symptoms, anxiety symptoms, poor wellbeing, and self-harm. Non-confounding variables were not included in models as, although there is evidence for their impact on mental health, there was limited support in the literature for these variables influencing patterns of social media use. All other demographic, mental health, prior social media use and social factors were determined to be confounders. However, parent mental health and family conflict were not measured across relevant waves and thus were not included as covariates in models. For models of self-harm, we also adjusted for self-harm measured at t-1. Sensitivity analyses were performed in which mental health problems (defined as the presence of at least one of high depressive symptoms, high anxiety symptoms, or poor wellbeing) at time t were included as an additional confounder. For models of self-harm, sensitivity analyses included the presence of at least one of high depressive symptoms, high anxiety symptoms, poor wellbeing, or self-harm.

Distribution of age

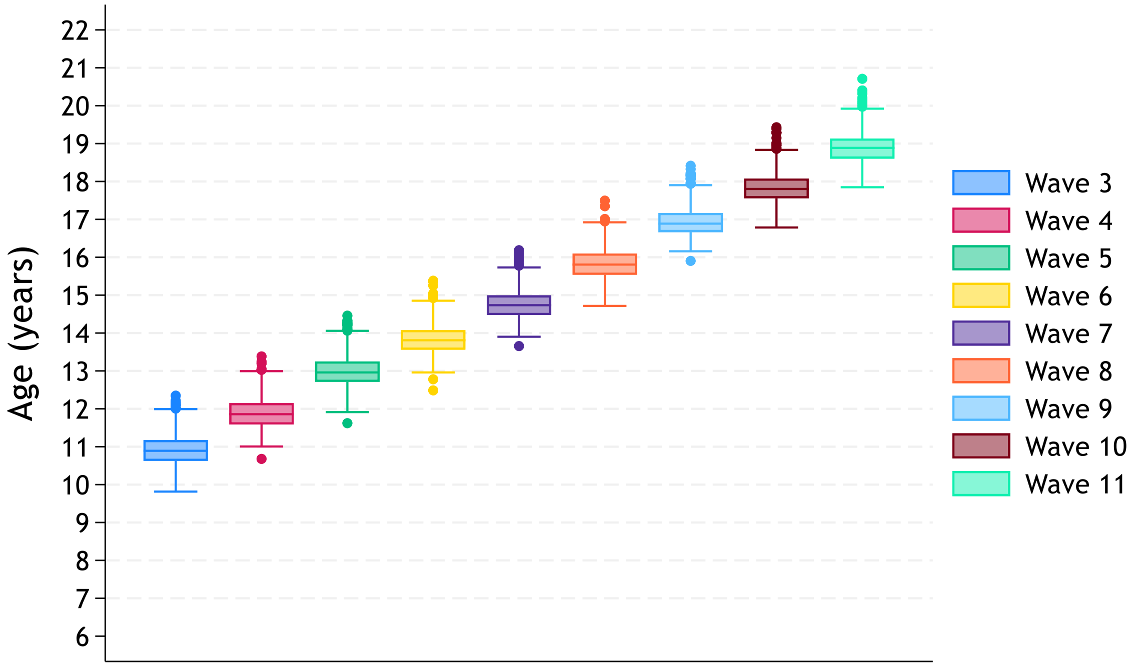

FIGURE S5: Distribution of age at each of waves 3 to 11.

Distribution of social media use

TABLE S4: Number (percentage) of study participants (N=1,195) in each social media use category (on a typical school day), at each of waves 4 to 10, by sex.

	**Wave 4**		**Wave 5**		**Wave 6**		**Wave 7**	
Social media use	Females	Males	Females	Males	Females	Males	Females	Males
<1 hr§	484 (86.6)	457 (91.6)	350 (67.0)	368 (80.7)	256 (49.1)	290 (61.7)	208 (44.1)	229 (56.1)
1-2 hrs§	48 (8.6)	30 (6.0)	89 (17.0)	57 (12.5)	142 (27.3)	107 (22.8)	159 (33.7)	98 (24.0)
>2 hrs	27 (4.8)	12 (2.4)	83 (15.9)	31 (6.8)	123 (23.6)	73 (15.5)	105 (22.2)	81 (19.9)
Missing†	84 (13.1)	53 (9.6)	121 (18.8)	96 (17.4)	122 (19.0)	82 (14.9)	171 (26.6)	144 (26.1)
	**Wave 8**		**Wave 9**		**Wave 10**			
Social media use	Females	Males	Females	Males	Females	Males		
<1 hr§	144 (31.2)	165 (433)	97 (20.7)	109 (29.8)	77 (16.7)	90 (25.3)		
1-2 hrs§	143 (31.0)	114 (29. 9)	146 (31.1)	123 (33.6)	147 (31.8)	135 (37.9)		
>2 hrs	174 (37.7)	102 (26.8)	226 (48.2)	134 (36.6)	238 (51.5)	131 (36.8)		
Missing†	182 (28.3)	171 (31.0)	174 (27.1)	186 (33.7)	181 (28.1)	196 (35.5)		

§ Number of individuals (percent of non-missing responses).

† Number of individuals (percent of total sample).

Distribution of outcome measures

TABLE S5: Number (percentage) of study participants (N=1,195) reporting high depressive symptoms, high anxiety symptoms, and poor general wellbeing, at each of waves 5 to 11, by sex.

	Wave 5		Wave 6		Wave 7		Wave 8	
**Depressive symptoms**	**Females**	**Males**	**Females**	**Males**	**Females**	**Males**	Females	Males
No§	441 (88.9)	418 (95.9)	419 (81.2)	435 (94.6)	370 (78.9)	384 (94.3)	312 (68.4)	344 (91.2)
Yes§	55 (11.1)	18 (4.1)	97 (18.8)	25 (5.4)	99 (21.1)	23 (5.7)	144 (31.6)	33 (8.8)
Missing†	147 (22.9)	116 (21.0)	127 (19.8)	92 (16.7)	174 (27.1)	145 (26.3)	187 (29.1)	175 (31.7)
	**Wave 9**		**Wave 10**		**Wave 11**			
**Depressive symptoms**	Females	Males	Females	Males	Females	Males		
No§	251 (53.9)	315 (86·.5)	238 (52.0)	289 (81.4)	288 (61.4)	293 (83.7)		
Yes§	215 (46.1)	49 (13.5)	220 (48.0)	66 (18.6)	181 (38.6)	57 (16.3)		
Missing†	177 (27.5)	188 (34.1)	185 (28.8)	197 (35.7)	174 (27.1)	202 (36.6)		
	**Wave 5**		**Wave 6**		**Wave 7**		**Wave 8**	
**Anxiety symptoms**	Females	Males	Females	Males	Females	Males	Females	Males
No§	468 (89.7)	443 (96.5)	440 (84.3)	446 (95.7)	377 (79.9)	389 (94.6)	329 (71.7)	352 (91.4)
Yes§	54 (10.3)	16 (3.5)	82 (15.7)	20 (4.3)	95 (20.1)	22 (5.4)	130 (28.3)	33 (8.6)
Missing†	121 (18.8)	93 (16.3)	121 (18.8)	86 (15.6)	171 (26.6)	141 (25.5)	184 (28.6)	167 (30.3)
	**Wave 9**		**Wave 10**		**Wave 11**			
**Anxiety symptoms**	Females	Males	Females	Males	Females	Males		
No§	305 (65.3)	325 (88.6)	284 (61.7)	304 (85.9)	294 (62.4)	298 (84.9)		
Yes§	162 (34.7)	42 (11.4)	176 (38.3)	50 (14.1)	177 (37.6)	53 (15.1)		
Missing†	176 (27.4)	185 (33.5)	183 (28.5)	198 (35.9)	172 (26.7)	201 (36.4)		
	**Wave 5**		**Wave 6**		**Wave 7**		**Wave 8**	
**Poor wellbeing**	Females	Males	Females	Males	Females	Males	**Females**	**Males**
No§	422 (80.4)	399 (86.6)	368 (70.1)	390 (83.0)	351 (73.9)	344 (83.5)	318 (68.5)	316 (82.3)
Yes§	103 (19.6)	62 (13.4)	157 (29.9)	80 (17.0)	124 (26.1)	68 (16.5)	146 (31.5)	68 (17.7)

Missing†	118 (18.4)	91 (16.5)	118 (18.4)	82 (14.9)	168 (26.1)	140 (25.4)	179 (27.8)	168 (30.4)
	**Wave 9**		**Wave 10**					
**Poor wellbeing**	**Females**	**Males**	**Females**	**Males**				
No§	243 (51.7)	255 (69.5)	209 (45.2)	248 (69.3)				
Yes§	227 (48.3)	112 (30.5)	253 (54.8)	110 (30.7)				
Missing†	173 (26.9)	185 (33.5)	181 (28.1)	194 (35.1)				
	**Wave 5**		**Wave 6**		**Wave 7**		**Wave 8**	
**Self-harm**	**Females**	**Males**	**Females**	**Males**	**Females**	**Males**	**Females**	**Males**
No§	492 (96.5)	442 (96.7)	479 (94.7)	448 (97.8)	434 (93.3)	399 (97.3)	388 (86.2)	367 (97.1)
Yes§	18 (3.5)	15 (3.3)	27 (5.3)	10 (2.2)	31 (6.7)	11 (2.7)	62 (13.8)	11 (2.9)
Missing†	133 (20.7)	95 (17.2)	137 (21.3)	94 (17.0)	178 (27.7)	142 (25.7)	193 (30.0)	174 (31.5)
	**Wave 9**		**Wave 10**		**Wave 11**			
**Self-harm**	**Females**	**Males**	**Females**	**Males**	**Females**	**Males**		
No§	401 (87.2)	341 (95.3)	380 (84.6)	337 (95.7)	407 (88.7)	336 (96.3)		
Yes§	59 (12.8)	17 (4.7)	69 (15.4)	15 (4.3)	52 (11.3)	13 (3.7)		
Missing†	183 (28.5)	194 (35.1)	194 (30.2)	200 (36.2)	184 (28.6)	203 (36.8)		

§ Number of individuals (percent of non-missing responses).

† Number of individuals (percent of total sample).

Estimated risk ratios and risk differences of social media use on future mental health

TABLE S6: Estimated risk ratios (95% CI) and risk differences (95% CI) of social media use (on a typical week day) on future mental health (high depressive symptoms, high anxiety symptoms, poor wellbeing, self-harm), over adolescence, overall and by sex.

		RR (95%CI)				RD* (95%CI)		
**Depressive symptoms**		**Overall**	**Females**	**Males**		**Overall**	**Females**	**Males**
1-2 hrs vs <1 hr SMU		1.03 (0.91, 1.18)	1.04 (0.90, 1.20)	1.01 (0.75, 1.36)		0.7 (-2.2, 3.6)	1.2 (-3.2, 5.6)	0.2 (-3.3, 3.7)
>2 hrs vs <1 hr SMU		1.29 (1.12, 1.49)	1.22 (1.06, 1.41)	1.49 (1.10, 2.02)		6.3 (2.7, 9.9)	6.7 (2.0, 11.4)	5.8 (1.1, 10.5)
>2 hrs vs 1-2 hrs SMU		1.25 (1.09, 1.43)	1.18 (1.03, 1.34)	1.47 (1.07, 2.02)		5.6 (2.1, 9.0)	5.5 (1.2, 9.9)	5.6 (0.9, 10.3)
**Anxiety symptoms**								
1-2 hrs vs <1 hr SMU		1.00 (0.87, 1.14)	0.94 (0.81, 1.08)	1.19 (0.86, 1.64)		-0.1 (-2.9, 2.7)	-1.8 (-5.8, 2.3)	1.9 (-1.7, 5.4)
>2 hrs vs <1 hr SMU		1.11 (0.94, 1.30)	0.98 (0.83, 1.16)	1.52 (1.09, 2.13)		2.1 (-1.3, 5.6)	-0.5 (-5.3, 4.3)	5.2 (0.8, 9.6)
>2 hrs vs 1-2 hrs SMU		1.11 (0.96, 1.29)	1.05 (0.90, 1.22)	1.28 (0.94, 1.76)		2.2 (-0.9, 5.4)	1.2 (-3.0, 5.5)	3.4 (-0.9, 7.7)
**Poor wellbeing**								
1-2 hrs vs <1 hr SMU		1.12 (1.01, 1.24)	1.17 (1.04, 1.31)	1.03 (0.85, 1.25)		3.3 (0.2, 6.4)	5.6 (1.2, 10.0)	0.7 (-3.7, 5.1)
>2 hrs vs <1 hr SMU		1.17 (1.04, 1.32)	1.19 (1.04, 1.37)	1.13 (0.90, 1.41)		4.9 (1.1, 8.6)	6.5 (1.5, 11.6)	2.9 (-2.5, 8.4)
>2 hrs vs 1-2 hrs SMU		1.05 (0.93, 1.18)	1.02 (0.90, 1.17)	1.10 (0.87, 1.38)		1.5 (-2.3, 5.4)	0.9 (-4.3, 6.1)	2.2 (-3.3, 7.8)
**Self-harm**								
1-2 hrs vs <1 hr SMU		1.06 (0.82, 1.37)	1.17 (0.88, 1.56)	0.83 (0.50, 1.38)		0.5 (-1.9, 3.0)	2.0 (-1.6, 5.6)	-1.1 (-4.3, 2.0)
>2 hrs vs <1 hr SMU		1.23 (0.94, 1.61)	1.15 (0.84, 1.56)	1.37 (0.86, 2.20)		2.1 (-0.7, 5.0)	1.7 (-2.1, 5.5)	2.6 (-1.3, 6.6)
>2 hrs vs 1-2 hrs SMU		1.16 (0.91, 1.47)	0.98 (0.76, 1.26)	1.65 (1.01, 2.69)		1.6 (-1.0, 4.2)	-0.3 (-3.7, 3.1)	3.8 (0.0, 7.5)

* Risk differences represented as per 100.

TABLE S7: Estimated risk ratios (95% CI) and risk differences (95% CI) of social media use (on a typical week day) on future mental health (high depressive symptoms, high anxiety symptoms, poor wellbeing, self-harm), by period of adolescence and by sex.

	RR (95%CI)			RD* (95%CI)	
**Depressive symptoms**	**Females**	**Males**		**Females**	**Males**
Early adolescence					
1-2 hrs vs <1 hr SMU	1.26 (0.88, 1.80)	1.26 (0.77, 2.06)		3.9 (-2.5, 10.3)	1.6 (-2.1, 5.3)
>2 hrs vs <1 hr SMU	1.72 (1.22, 2.43)	2.18 (1.32, 3.59)		10.8 (2.7, 18.9)	7.2 (0.7, 13.7)
>2 hrs vs 1-2 hrs SMU	1.37 (0.89, 2.11)	1.73 (0.94, 3.18)		6.9 (-2.8, 16.5)	5.6 (-1.2, 12.4)
Mid adolescence					
1-2 hrs vs <1 hr SMU	1.00 (0.85, 1.18)	0.97 (0.69, 1.36)		0.1 (-5.2, 5.4)	-0.3 (-4.2, 3.6)
>2 hrs vs <1 hr SMU	1.14 (0.97, 1.35)	1.38 (1.00, 1.90)		4.8 (-1.1, 10.7)	4.5 (-0.2, 9.3)
>2 hrs vs 1-2 hrs SMU	1.14 (0.97, 1.34)	1.42 (1.00, 2.02)		4.7 (-1.1, 10.5)	4.9 (-0.1, 9.8)
Late adolescence					
1-2 hrs vs <1 hr SMU	1.00 (0.82, 1.22)	0.97 (0.70, 1.35)		0.1 (-8.2, 8.3)	-0.5 (-6.0, 5.1)
>2 hrs vs <1 hr SMU	1.13 (0.94, 1.36)	1.37 (0.98, 1.90)		5.5 (-2.3, 13.3)	6.3 (-0.2, 12.8)
>2 hrs vs 1-2 hrs SMU	1.13 (0.97, 1.32)	1.41 (1.02, 1.93)		5.5 (-1.0, 12.0)	6.8 (0.5, 13.0)
**Anxiety symptoms**					
Early adolescence					
1-2 hrs vs <1 hr SMU	1.22 (0.85, 1.75)	1.66 (1.02, 2.69)		3.0 (-2.8, 8.7)	3.2 (-0.6, 7.0)
>2 hrs vs <1 hr SMU	1.36 (0.91, 2.05)	2.32 (1.38, 3.88)		5.0 (-2.1, 12.0)	6.5 (0.7, 12.3)
>2 hrs vs 1-2 hrs SMU	1.12 (0.70, 1.79)	1.40 (0.79, 2.47)		2.0 (-6.2, 10.2)	3.3 (-2.6, 9.1)
Mid adolescence					
1-2 hrs vs <1 hr SMU	0.90 (0.75, 1.08)	1.14 (0.79, 1.66)		-2.9 (-8.1, 2.3)	1.5 (-2.6, 5.5)
>2 hrs vs <1 hr SMU	0.97 (0.80, 1.18)	1.53 (1.07, 2.17)		-0.8 (-6.6, 4.9)	5.4 (0.6, 10.1)
>2 hrs vs 1-2 hrs SMU	1.08 (0.88, 1.31)	1.34 (0.92, 1.94)		2.1 (-3.6, 7.7)	3.9 (-1.2, 9.0)
Late adolescence					
1-2 hrs vs <1 hr SMU	0.89 (0.73, 1.08)	1.07 (0.74, 1.55)		-4.8 (-12.8, 3.3)	1.1 (-4.5, 6.6)
>2 hrs vs <1 hr SMU	0.87 (0.71, 1.06)	1.26 (0.86, 1.83)		-5.6 (-13.7, 2.6)	3.8 (-2.4, 9.9)

>2 hrs vs 1-2 hrs SMU	0.98 (0.83, 1.16)	1.17 (0.84, 1.62)		-0.8 (-7.1, 5.4)	2.7 (-2.9, 8.3)
**Poor wellbeing**					
Early adolescence					
1-2 hrs vs <1 hr SMU	1.34 (1.08, 1.66)	1.17 (0.87, 1.59)		8.2 (1.6, 14.9)	2.9 (-2.8, 8.7)
>2 hrs vs <1 hr SMU	1.43 (1.13, 1.82)	1.37 (0.98, 1.91)		10.6 (2.8, 18.4)	6.2 (-1.1, 13.4)
>2 hrs vs 1-2 hrs SMU	1.07 (0.81, 1.42)	1.17 (0.79, 1.72)		2.3 (-7.1, 11.8)	3.2 (-5.0, 11.4)
Mid adolescence					
1-2 hrs vs <1 hr SMU	1.10 (0.94, 1.28)	0.95 (0.76, 1.17)		3.2 (-2.2, 8.7)	-1.2 (-6.2, 3.7)
>2 hrs vs <1 hr SMU	1.14 (0.96, 1.34)	1.06 (0.83, 1.34)		4.6 (-1.3, 10.5)	1.4 (-4.5, 7.3)
>2 hrs vs 1-2 hrs SMU	1.04 (0.88, 1.22)	1.12 (0.87, 1.43)		1.4 (-4.7, 7.5)	2.7 (-3.3, 8.6)
Late adolescence					
1-2 hrs vs <1 hr SMU	1.15 (0.96, 1.37)	1.06 (0.80, 1.41)		7.5 (-2.0, 17.0)	2.0 (-7.0, 10.9)
>2 hrs vs <1 hr SMU	1.08 (0.91, 1.29)	1.03 (0.78, 1.36)		4.2 (-4.9, 13.4)	0.9 (-7.8, 9.6)
>2 hrs vs 1-2 hrs SMU	0.94 (0.82, 1.09)	0.97 (0.75, 1.25)		-3.2 (-11.3, 4.8)	-1.0 (-9.3, 7.2)
**Self-harm**					
Early adolescence					
1-2 hrs vs <1 hr SMU	1.42 (0.61, 3.33)	0.97 (0.36, 2.61)		2.3 (-3.5, 8.1)	0.1 (-4.2, 4.3)
>2 hrs vs <1 hr SMU	2.26 (1.12, 4.57)	2.62 (1.19, 5.77)		6.6 (-0.1, 13.2)	7.1 (-1.3, 15.4)
>2 hrs vs 1-2 hrs SMU	1.59 (0.61, 4.11)	2.69 (0.96, 7.52)		4.3 (-4.3, 12.9)	7.0 (-1.2, 15.2)
Mid adolescence					
1-2 hrs vs <1 hr SMU	1.21 (0.85, 1.73)	0.85 (0.51, 1.42)		2.5 (-2.2, 7.3)	-1.2 (-4.9, 2.6)
>2 hrs vs <1 hr SMU	1.15 (0.78, 1.68)	1.31 (0.80, 2.17)		1.7 (-3.2, 6.7)	2.6 (-2.2, 7.4)
>2 hrs vs 1-2 hrs SMU	0.95 (0.67, 1.34)	1.55 (0.90, 2.66)		-0.8 (-5.7, 4.1)	3.8 (-0.9, 8.5)
Late adolescence					
1-2 hrs vs <1 hr SMU	1.08 (0.70, 1.66)	0.75 (0.38, 1.46)		1.1 (-5.2, 7.3)	-1.7 (-5.7, 2.3)
>2 hrs vs <1 hr SMU	0.95 (0.61, 1.47)	1.08 (0.59, 1.98)		-0.8 (-6.9, 5.4)	0.5 (-3.6, 4.7)
>2 hrs vs 1-2 hrs SMU	0.88 (0.62, 1.24)	1.44 (0.78, 2.66)		-1.8 (-6.8, 3.2)	2.2 (-1.5, 6.0)

Sensitivity analysis: Estimated risk differences and risk ratios of social media use on future mental health, after inclusion of a measure of the presence of any mental health problems measured at same wave as exposure

TABLE S8: Estimated risk ratios (95% CI) and risk differences (95% CI) of social media use (on a typical week day) on future mental health (high depressive symptoms, high anxiety symptoms, poor wellbeing, self-harm), over adolescence, overall and by sex.

		RR (95%CI)				RD* (95%CI)		
**Depressive symptoms**		**Overall**	**Females**	**Males**		**Overall**	**Females**	**Males**
1-2 hrs vs <1 hr SMU		0.99 (0.87, 1.13)	0.99 (0.87, 1.14)	0.99 (0.74, 1.34)		-0.1 (-2.9, 2.7)	-0.2 (-4.4, 4.0)	-0.1 (-3.5, 3.4)
>2 hrs vs <1 hr SMU		1.22 (1.07, 1.41)	1.16 (1.01, 1.33)	1.43 (1.06, 1.94)		5.0 (1.5, 8.5)	4.9 (0.3, 9.4)	5.1 (0.6, 9.6)
>2 hrs vs 1-2 hrs SMU		1.23 (1.08, 1.41)	1.16 (1.03, 1.32)	1.45 (1.05, 1.98)		5.1 (1.8, 8.4)	5.1 (0.9, 9.2)	5.2 (0.6, 9.7)
**Anxiety symptoms**								
1-2 hrs vs <1 hr SMU		0.95 (0.83, 1.09)	0.90 (0.78, 1.03)	1.16 (0.83, 1.60)		-0.9 (-3.7, 1.8)	-3.1 (-7.0, 0.8)	1.6 (-2.0, 5.1)
>2 hrs vs <1 hr SMU		1.04 (0.89, 1.21)	0.92 (0.79, 1.08)	1.45 (1.04, 2.01)		0.8 (-2.5, 4.1)	-2.4 (-6.9, 2.1)	4.5 (0.3, 8.7)
>2 hrs vs 1-2 hrs SMU		1.09 (0.94, 1.25)	1.03 (0.89, 1.19)	1.25 (0.92, 1.71)		1.7 (-1.2, 4.7)	0.7 (-3.3, 4.7)	2.9 (-1.2, 7.0)
**Poor wellbeing**								
1-2 hrs vs <1 hr SMU		1.07 (0.97, 1.19)	1.09 (0.98, 1.23)	1.04 (0.86, 1.25)		2.2 (-0.9, 5.2)	3.3 (-0.9, 7.5)	0.9 (-3.4, 5.2)
>2 hrs vs <1 hr SMU		1.09 (0.98, 1.23)	1.10 (0.97, 1.26)	1.08 (0.88, 1.32)		2.8 (-0.7, 6.3)	3.6 (-1.2, 8.4)	1.8 (-3.2, 6.7)
>2 hrs vs 1-2 hrs SMU		1.02 (0.91, 1.14)	1.01 (0.89, 1.15)	1.04 (0.84, 1.28)		0.6 (-3.0, 4.1)	0.3 (-4.6, 5.3)	0.9 (-4.2, 5.9)
**Self-harm**								
1-2 hrs vs <1 hr SMU		1.00 (0.78, 1.29)	1.09 (0.83, 1.44)	0.83 (0.50, 1.38)		0.0 (-2.4, 2.5)	1.1 (-2.4, 4.6)	-1.2 (-4.4, 2.0)
>2 hrs vs <1 hr SMU		1.12 (0.86, 1.46)	1.05 (0.78, 1.41)	1.28 (0.80, 2.06)		1.2 (-1.5, 4.0)	0.5 (-3.2, 4.3)	2.0 (-1.8, 5.9)
>2 hrs vs 1-2 hrs SMU		1.12 (0.88, 1.42)	0.96 (0.75, 1.22)	1.55 (0.94, 2.56)		1.2 (-1.3, 3.7)	-0.6 (-3.8, 2.7)	3.2 (-0.4, 6.9)

* Risk differences represented as per 100.

TABLE S9: Estimated risk ratios (95% CI) and risk differences (95% CI) of social media use (on a typical week day) on future mental health (high depressive symptoms, high anxiety symptoms, poor wellbeing, self-harm), by period of adolescence and by sex.

	**RR (95%CI)**			**RD* (95%CI)**	
**Depressive symptoms**	**Females**	**Males**		**Females**	**Males**
Early adolescence					
1-2 hrs vs <1 hr SMU	1.16 (0.82, 1.66)	1.20 (0.72, 1.99)		2.5 (-3.5, 8.6)	1.3 (-2.4, 4.9)
>2 hrs vs <1 hr SMU	1.56 (1.11, 2.20)	2.02 (1.22, 3.34)		8.5 (0.9, 16.2)	6.2 (0.2, 12.2)
>2 hrs vs 1-2 hrs SMU	1.34 (0.87, 2.07)	1.68 (0.91, 3.12)		6.0 (-3.0, 15.0)	5.0 (-1.4, 11.3)
Mid adolescence					
1-2 hrs vs <1 hr SMU	0.95 (0.81, 1.11)	0.93 (0.66, 1.31)		-1.6 (-6.8, 3.6)	-0.8 (-4.6, 3.1)
>2 hrs vs <1 hr SMU	1.09 (0.92, 1.27)	1.32 (0.96, 1.83)		2.9 (-2.8, 8.6)	3.9 (-0.8, 8.6)
>2 hrs vs 1-2 hrs SMU	1.14 (0.97, 1.34)	1.42 (0.99, 2.02)		4.5 (-1.1, 10.1)	4.6 (-0.2, 9.4)
Late adolescence					
1-2 hrs vs <1 hr SMU	0.98 (0.81, 1.19)	0.98 (0.71, 1.35)		-0.7 (-8.8, 7.4)	-0.4 (-5.9, 5.1)
>2 hrs vs <1 hr SMU	1.10 (0.92, 1.31)	1.34 (0.97, 1.85)		4.2 (-3.5, 11.9)	5.8 (-0.6, 12.1)
>2 hrs vs 1-2 hrs SMU	1.12 (0.97, 1.29)	1.37 (1.00, 1.87)		4.9 (-1.4, 11.2)	6.2 (0.1, 12.2)
**Anxiety symptoms**					
Early adolescence					
1-2 hrs vs <1 hr SMU	1.13 (0.79, 1.62)	1.61 (0.98, 2.64)		1.9 (-3.6, 7.4)	3.0 (-0.8, 6.7)
>2 hrs vs <1 hr SMU	1.20 (0.80, 1.80)	2.12 (1.26, 3.58)		2.9 (-3.7, 9.4)	5.5 (0.3, 10.7)
>2 hrs vs 1-2 hrs SMU	1.06 (0.67, 1.69)	1.32 (0.74, 2.35)		1.0 (-6.6, 8.5)	2.5 (-2.9, 8.0)
Mid adolescence					
1-2 hrs vs <1 hr SMU	0.85 (0.71, 1.02)	1.09 (0.75, 1.59)		-4.7 (-9.8, 0.4)	0.9 (-3.1, 5.0)
>2 hrs vs <1 hr SMU	0.91 (0.76, 1.10)	1.45 (1.02, 2.06)		-2.8 (-8.3, 2.8)	4.7 (0.0, 9.3)
>2 hrs vs 1-2 hrs SMU	1.07 (0.88, 1.30)	1.33 (0.91, 1.94)		1.9 (-3.5, 7.3)	3.7 (-1.2, 8.6)
Late adolescence					
1-2 hrs vs <1 hr SMU	0.87 (0.72, 1.06)	1.07 (0.74, 1.55)		-5.7 (-13.6, 2.3)	1.1 (-4.5, 6.6)
>2 hrs vs <1 hr SMU	0.84 (0.69, 1.02)	1.22 (0.85, 1.77)		-7.0 (-14.9, 0.9)	3.3 (-2.7, 9.2)
>2 hrs vs 1-2 hrs SMU	0.97 (0.82, 1.14)	1.14 (0.83, 1.57)		-1.3 (-7.4, 4.8)	2.2 (-3.1, 7.6)

**Poor wellbeing**					
Early adolescence					
1-2 hrs vs <1 hr SMU	1.24 (0.99, 1.55)	1.18 (0.87, 1.60)		6.0 (-0.6, 12.6)	3.1 (-2.8, 8.9)
>2 hrs vs <1 hr SMU	1.25 (0.98, 1.59)	1.23 (0.89, 1.71)		6.3 (-1.0, 13.7)	3.9 (-2.7, 10.5)
>2 hrs vs 1-2 hrs SMU	1.01 (0.76, 1.35)	1.04 (0.71, 1.53)		0.3 (-8.7, 9.3)	0.8 (-6.9, 8.6)
Mid adolescence					
1-2 hrs vs <1 hr SMU	1.02 (0.88, 1.19)	0.95 (0.77, 1.17)		0.8 (-4.6, 6.1)	-1.2 (-6.0, 3.7)
>2 hrs vs <1 hr SMU	1.06 (0.91, 1.24)	1.03 (0.82, 1.29)		2.3 (-3.4, 7.9)	0.7 (-4.8, 6.2)
>2 hrs vs 1-2 hrs SMU	1.04 (0.89, 1.22)	1.08 (0.86, 1.36)		1.5 (-4.3, 7.3)	1.9 (-3.7, 7.4)
Late adolescence					
1-2 hrs vs <1 hr SMU	1.10 (0.94, 1.30)	1.09 (0.83, 1.42)		5.4 (-3.5, 14.3)	2.7 (-5.7, 11.0)
>2 hrs vs <1 hr SMU	1.04 (0.89, 1.23)	1.02 (0.78, 1.32)		2.2 (-6.4, 10.9)	0.6 (-7.5, 8.6)
>2 hrs vs 1-2 hrs SMU	0.94 (0.82, 1.08)	0.94 (0.74, 1.19)		-3.2 (-10.8, 4.4)	-2.1 (-9.8, 5.6)
**Self-harm**					
Early adolescence					
1-2 hrs vs <1 hr SMU	1.26 (0.54, 2.91)	0.93 (0.34, 2.51)		1.5 (-3.9, 6.9)	-0.1 (-4.3, 4.0)
>2 hrs vs <1 hr SMU	1.87 (0.93, 3.78)	2.26 (1.02, 5.03)		4.8 (-1.1, 10.8)	5.6 (-1.8, 13.0)
>2 hrs vs 1-2 hrs SMU	1.49 (0.58, 3.81)	2.45 (0.87, 6.90)		3.3 (-4.4, 11.1)	5.8 (-1.6, 13.1)
Mid adolescence					
1-2 hrs vs <1 hr SMU	1.11 (0.78, 1.57)	0.83 (0.50, 1.39)		1.4 (-3.3, 6.1)	-1.3 (-5.1, 2.4)
>2 hrs vs <1 hr SMU	1.03 (0.71, 1.50)	1.22 (0.74, 2.02)		0.4 (-4.5, 5.3)	1.9 (-2.8, 6.5)
>2 hrs vs 1-2 hrs SMU	0.93 (0.66, 1.31)	1.47 (0.85, 2.53)		-1.0 (-5.7, 3.7)	3.2 (-1.4, 7.8)
Late adolescence					
1-2 hrs vs <1 hr SMU	1.04 (0.68, 1.59)	0.77 (0.39, 1.53)		0.5 (-5.9, 6.9)	-1.5 (-5.6, 2.6)
>2 hrs vs <1 hr SMU	0.91 (0.59, 1.41)	1.07 (0.58, 1.98)		-1.4 (-7.7, 4.9)	0.5 (-3.7, 4.6)
>2 hrs vs 1-2 hrs SMU	0.88 (0.63, 1.23)	1.39 (0.75, 2.57)		-1.9 (-6.9, 3.1)	2.0 (-1.7, 5.7)

* Risk differences represented as per 100.

STROBE Statement—checklist of items that should be included in reports of observational studies

	Item No	Recommendation
**Title and abstract**	1	(a) Indicate the study's design with a commonly used term in the title or the abstract [Yes]
		(b) Provide in the abstract an informative and balanced summary of what was done and what was found [Yes]
**Introduction**		
Background/rationale	2	Explain the scientific background and rationale for the investigation being reported [Yes]
	3	State specific objectives, including any prespecified hypotheses [Yes
**Methods**		
Study design	4	Present key elements of study design early in the paper [Yes]
Setting	5	Describe the setting, locations, and relevant dates, including periods of recruitment, exposure, follow-up, and data collection [Yes (Supplementary Figure S1)]
Participants	6	(a) Cohort study—Give the eligibility criteria, and the sources and methods of selection of participants. Describe methods of follow-up [Yes]<br><br>Case-control study—Give the eligibility criteria, and the sources and methods of case ascertainment and control selection. Give the rationale for the choice of cases and controls [N/A]

		Cross-sectional study—Give the eligibility criteria, and the sources and methods of selection of participants [N/A]
		(b) Cohort study—For matched studies, give matching criteria and number of exposed and unexposed [N/A]<br>Case-control study—For matched studies, give matching criteria and the number of controls per case [N/A]
Variables	7	Clearly define all outcomes, exposures, predictors, potential confounders, and effect modifiers. Give diagnostic criteria, if applicable [Yes (Supplementary Table S3)]
Data sources/ measurement	8*	For each variable of interest, give sources of data and details of methods of assessment (measurement). Describe comparability of assessment methods if there is more than one group [Yes (Supplementary Table S3)]
Bias	9	Describe any efforts to address potential sources of bias [Yes Supplementary Figure S4]
Study size	10	Explain how the study size was arrived at [Yes]
Quantitative variables	11	Explain how quantitative variables were handled in the analyses. If applicable, describe which groupings were chosen and why [Yes (Supplementary Table S3)]
Statistical methods	12	(a) Describe all statistical methods, including those used to control for confounding [Yes]
		(b) Describe any methods used to examine subgroups and interactions [Yes]
		(c) Explain how missing data were addressed [Yes]
		(d) Cohort study—If applicable, explain how loss to follow-up was addressed [Yes]

		Case-control study—If applicable, explain how matching of cases and controls was addressed [N/A]
		Cross-sectional study—If applicable, describe analytical methods taking account of sampling strategy [N/A]
		(e) Describe any sensitivity analyses [Yes]
**Results**		
Participants	13*	(a) Report numbers of individuals at each stage of study—eg numbers potentially eligible, examined for eligibility, confirmed eligible, included in the study, completing follow-up, and analysed [Yes (Supplementary Figure S1)].
		(b) Give reasons for non-participation at each stage [not available - the non-participation at one wave did not preclude the participation at a future wave]
		(c) Consider use of a flow diagram [NA]
Descriptive data	14*	(a) Give characteristics of study participants (eg., demographic, clinical, social) and information on exposures and potential confounders [Yes]
		(b) Indicate number of participants with missing data for each variable of interest [Yes (Supplementary Table S2)]
		(c) Cohort study—Summarise follow-up time (eg, average and total amount) [Yes]
Outcome data	15*	Cohort study—Report numbers of outcome events or summary measures over time [Yes (Figure 2 and Supplementary Table S5)]
		Case-control study—Report numbers in each exposure category, or summary measures of exposure [N/A]
		Cross-sectional study—Report numbers of outcome events or summary measures [N/A]

Main results	16	(a) Give unadjusted estimates and, if applicable, confounder-adjusted estimates and their precision (eg, 95% confidence interval). Make clear which confounders were adjusted for and why they were included [We do not provide unadjusted estimates. The paper focuses on causal relationships; hence, the models need to include potential confounders due to our causal assumptions. See Supplementary Tables S6 & S7 for the confounder-adjusted estimates and their precision]
		(b) Report category boundaries when continuous variables were categorized [Yes (Supplementary Table S3)]
		(c) If relevant, consider translating estimates of relative risk into absolute risk for a meaningful time period [Yes – risk differences have been reported (along with risk ratios)]
Other analyses	17	Report other analyses done—eg analyses of subgroups and interactions, and sensitivity analyses [Yes (Supplementary Tables S8 & S9)]
**Discussion**		
Key results	18	Summarise key results with reference to study objectives [Yes]
Limitations	19	Discuss limitations of the study, taking into account sources of potential bias or imprecision. Discuss both direction and magnitude of any potential bias [Yes]
Interpretation	20	Give a cautious overall interpretation of results considering objectives, limitations, multiplicity of analyses, results from similar studies, and other relevant evidence [Yes]
Generalisability	21	Discuss the generalisability (external validity) of the study results [Yes
**Other information**		
Funding	22	Give the source of funding and the role of the funders for the present study and, if applicable, for the original study on which

the present article is based [Not included in the submitted manuscript due to blind review]

*Give information separately for cases and controls in case-control studies and, if applicable, for exposed and unexposed groups in cohort and cross-sectional studies.

Note: An Explanation and Elaboration article discusses each checklist item and gives methodological background and published examples of transparent reporting. The STROBE checklist is best used in conjunction with this article (freely available on the Web sites of PLoS Medicine at http://www.plosmedicine.org/, Annals of Internal Medicine at http://www.annals.org/, and Epidemiology at http://www.epidem.com/). Information on the STROBE Initiative is available at www.strobe-statement.org.

Note: The page numbers in this checklist refer to the submitted manuscript, not to the published article.

Supplementary Material Reference List

Australian Bureau of Statistics. Census of Population and Housing: Socio-Economic Indexes for Areas (SEIFA), Australia, 2011 [Internet]. Available at: https://www.abs.gov.au/ausstats/abs@.nsf/DetailsPage/2033.0.55.0012011 (accessed Sept 17, 2024).

Bond L, Wolfe S, Tollit M, et al. A comparison of the Gatehouse Bullying Scale and the Peer Relations Questionnaire for students in secondary school. *J School Health* 2007;77:75–79.

Kroenke K, Spitzer RL, Williams JBW, et al. Anxiety disorders in primary care: prevalence, impairment, comorbidity, and detection. *Ann Intern Med* 2007;146(5):317–325.

Moran P, Coffey C, Romaniuk H, et al. The natural history of self-harm from adolescence to young adulthood: a population-based cohort study. *Lancet* 2012;379(9812):236–243.

Rhew IC, Simpson K, Tracy M, et al. Criterion validity of the short mood and feelings questionnaire and one-and two item depression screens in young adolescents. *Child Adolesc Psychiatry Ment Health* 2010;4:8

Thabrew H, Stasiak K, Bavin LM, et al. Validation of the Mood and Feelings Questionnaire (MFQ) and Short Mood and Feelings Questionnaire (SMFQ) in New Zealand help-seeking adolescents. *Int J Methods Psychiatr Res* 2018;27(3):e1610.
